# Improved electrical and thermo-mechanical properties of a MWCNT/In–Sn–Bi composite solder reflowing on a flexible PET substrate

**DOI:** 10.1038/s41598-017-14263-6

**Published:** 2017-10-23

**Authors:** Sang Hoon Kim, Min-soo Park, Joon-Phil Choi, Clodualdo Aranas Jr.

**Affiliations:** 1 Powder Technology Department, Korea Institute of Materials Science, Changwon, 51508 Republic of Korea; 2 School of Materials Science and Engineering, Pusan National University, Busan, 46241 Republic of Korea; 3 Department of Mining and Materials Engineering, McGill University, 3610 University Street, Montreal, QC H3A 0C5 Canada

## Abstract

Multi-walled carbon nanotube (MWCNT)/indium–tin–bismuth (In–Sn–Bi) composite nanostructures in which In–Sn–Bi nanoparticles have been penetrated by the MWCNT arrays were synthesized using a chemical reduction method. The incorporation of 0.6 wt% MWCNTs with high electrical conductivity into the In-based solder resulted in low minimum electrical resistivity (19.9 ± 1.0 µΩ·cm). Despite being reflowed at the relatively low temperature of 110 °C, the composite solder nanostructures were able to form mechanically stable solder bumps on a flexible polyethylene terephthalate (PET) substrate due to the MWCNT arrays with a high thermal conductivity of 3000 W/(m·K) and In–Sn–Bi nanoparticles with a low melting temperature of 98.2 °C. Notably, the composite solder bumps exhibited high flexibility (17.7% resistance increase over 1000 cycles of operation in a bending test) and strong adhesion strength (0.9 N average shear strength in a scratch test) on the plastic substrate because of the presence of mechanically flexible and strong MWCNTs dispersed within the solder matrix materials. These overall properties are due to the improved diffusivity of the composite solder nanostructures by the cover of the In–Sn–Bi nanoparticles along the MWCNT arrays and the network structure formation of the composite solder bumps.

## Introduction

The ever-rising demand for slim, compact, and lightweight electronic components in flexible microelectronic packaging requires more advanced solder bumps with higher electrical and thermal performance, and stronger mechanical strength^1–8^. In this respect, Bi-based solder has been the primary choice for joining future conductive polymer-based microchips on plastic substrates; however, their insufficient electrical resistivity (38.3 µΩ·cm in the case of Bi–Sn solder), excessively high reflow temperature (139 °C), and inadequate mechanical strength (elastic modulus of 11.9 GPa) still constrain their use in the interconnectability of electronic components^3,9^. Meanwhile, conventional Sn-based soldering entails the use of high reflow temperatures of 180–250 °C, which exceeds the decomposition temperature of most plastic substrates used in flexible microelectronic packaging fields, and this may lead to the thermal stiffness of flexible plastic substrates used as printed circuit boards in the near future^10–13^. Furthermore, conventional solder adhesives often fail or detach due to the force of an external impact^14^. To address these problems, a more advanced solder needs to be developed that enables low-temperature joining of various temperature-sensitive electronic materials. In addition, such intrinsic limitations of conventional solders can also be overcome by using composite solders reinforced with carbon nanomaterials.

The incorporation of carbon nanomaterials with graphene structures can impart much more rapid electron transfer than conventional solders^15^. Subsequently, reinforcement with carbon nanomaterials having high thermal conductivity can be used to tailor a network structure to effectively transfer the outer thermal energy to the solder matrix^16^. Finally, metal matrix composites (MMCs) have superior mechanical performance, mostly due to reinforcement effects with strong mechanical resistance to external impact energy^3^. For example, Nai *et al*. have reported that widely explored MWCNTs can be utilized to revolutionize the transport of electric/electronic signal transmission of the solder, and as a result, may be suitable as reinforcement^17^. However, there is a major challenge for the employment of MWCNTs as a reinforcement material in a solder matrix due to high contact resistance (the Schottky barrier effect) at the interface between the MWCNT arrays and the solder constituents^18,19^. Hence, many studies have focused on their interfacial interactions because the difference in the work function between the MWCNT arrays and the solder constituents gives rise to an electron-transfer barrier that impedes electron tunneling, causing high contact resistance^18^. There is another challenge which must be overcome to increase the reactivity of the MWCNT arrays to the solder constituents; due to the chemical inertness of MWCNTs along with low reactivity and poor diffusivity, their surfaces bond weakly with solder constituents necessary for promoting the interconnectability of electronic components^19^. These issues have been severely restricting factors for many potential applications of composite solders (including carbon nanotubes) requiring good electrical conductivity and adhesive properties.

Herein for the first time, we investigate the synthesis and performance of MWCNT/In–Sn–Bi composite solder reflowed on a polyethylene terephthalate (PET) substrate. First, the ternary In–Sn–Bi nanoparticles were directly reinforced with monolithic (0.6 wt%) MWCNT arrays with high electrical and thermal conductivity. Next, we proved that the composite solder nanostructures can be applied for reflowing on the PET substrate due to its low melting temperature of 97.9 °C. During the reflow process, these composite solder nanostructures rapidly diffuse along the surface of the carbon nanotubes with high thermal conductivity, but in the absence of carbides, which indicates no surface damage of the carbon nanotubes. Finally, we demonstrated via bending and scratch tests that the formation of a network structure of composite solder bumps is a key mechanism in revealing their high flexibility and strong adhesive strength when reflowed on the flexible PET substrate.

## Results and Discussion

**Figure 1:**
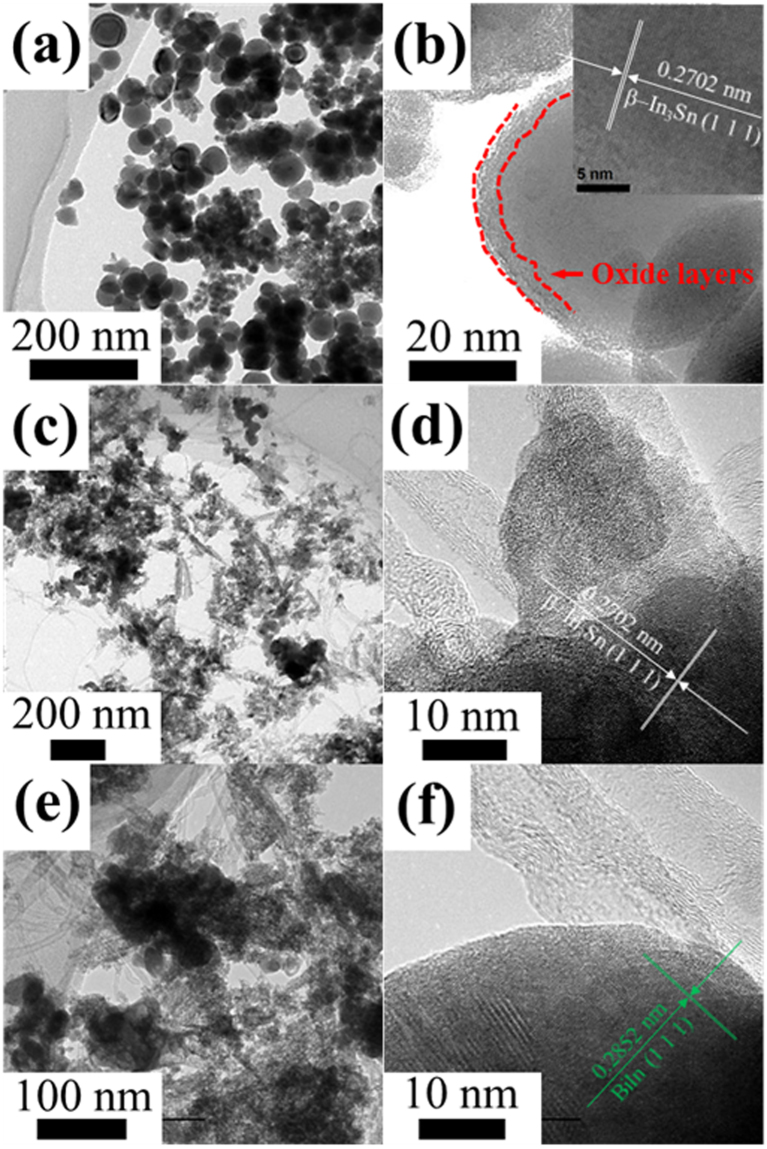
Low magnification TEM images and high resolution TEM images of In–Sn (**a**) and In–Sn–Bi nanoparticles (**b**), and 0.6MWCNT/In–Sn–Bi (**c** and **d**) and 1.2MWCNT/In–Sn–Bi composite nanostructures (**e** and **f**). High-resolution images of the tetragonal In_3_Sn (**d**) and BiIn (**f**) phases showing their (1 1 1) planes. The solder nanoparticles present a spherical shape, while the composite nanostructures exhibit the tubular shape of the MWCNTs embedded within the irregular shapes of the nanoparticles, which provided improvements in the solder’s electrical and thermo-mechanical properties.

A comparison between the as-synthesized In–Sn conventional and In–Sn–Bi reference nanoparticles and MWCNT/In–Sn–Bi composite nanostructures is provided in the TEM images shown in Fig. 1. More specifically, Fig. 1a contains a low-magnification TEM image of In–Sn nanoparticles disclosing their spherical shape and small size, while Fig. 1b is a high-resolution TEM image of the In–Sn–Bi nanoparticles with a lattice fringe measurement of In_3_Sn having a (1 1 1) plane and 0.2702 nm distance. Figure 1c and e present the MWCNT/In–Sn–Bi nanostructures in which the In–Sn–Bi nanoparticles having a broad size distribution were randomly grafted onto the MWCNT arrays with a tubular structure. That is to say, the cotton-ball-like solder nanostructures were embedded on the surface of the perforated MWCNT arrays. Figure 1d and f reveal clear lattice fringes in the composite solder nanostructures separated by 0.2702 and 0.2852 nm that correspond to the (1 1 1) planes of the tetragonal In_3_Sn and BiIn phases, respectively.

**Figure 2:**
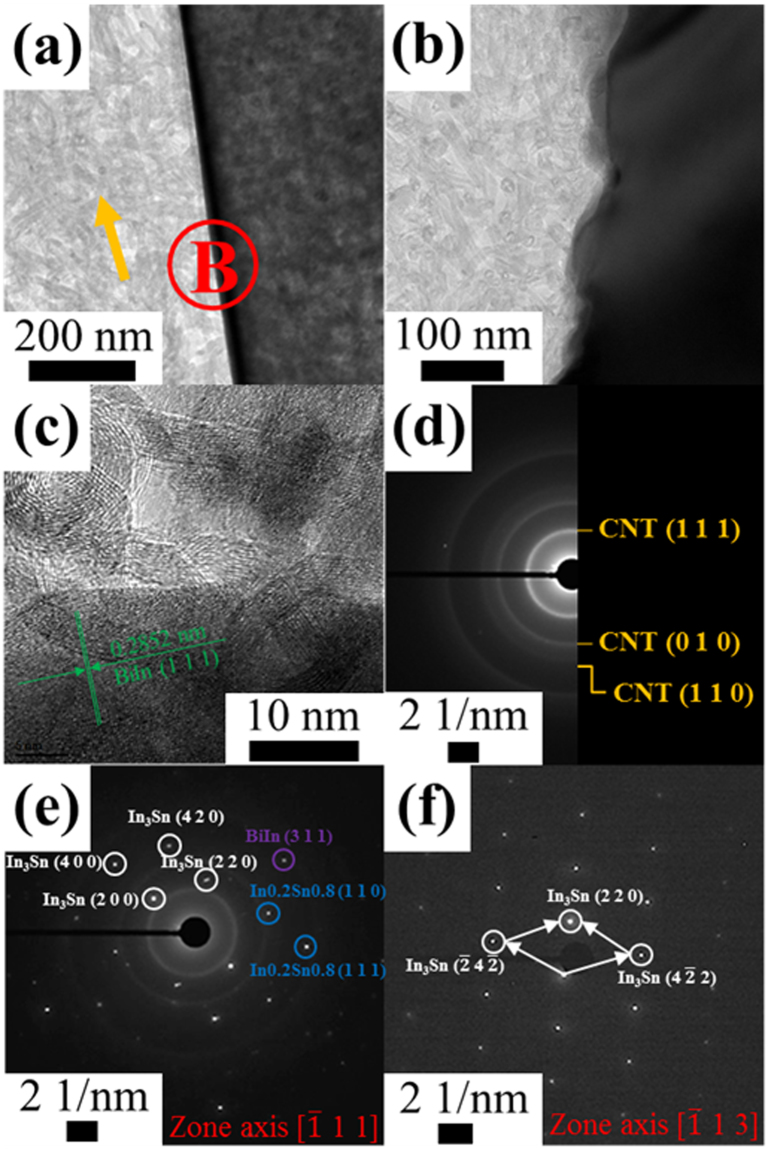
Low magnification TEM images (**a** and **b**), a high resolution TEM image (**c**), and SAED patterns of 0.6MWCNT/In–Sn–Bi composite solder after the reflow at 110 °C: the MWCNT region (**d**), the interfacial region between the two phases (**e**), and the metallic region (**f**). The separation between the two different phases (MWCNT arrays and solder constituents) of the composite solder increased the diffusivity and wettability of the composite solder bumps.

The network structure of the MWCNT/In–Sn–Bi composite solder prepared by the reflow process is shown in Fig. 2. As shown in Fig. 2a, the MWCNT/In–Sn–Bi composite solder has two different solder matrix phases with a sharp interfacial boundary, whereas Fig. 2b shows a composite solder with a rugged interfacial boundary. The high-resolution TEM image in Fig. 2c shows slightly bent MWCNTs, each with a disordered lattice fringe and a cluster of solder nanostructures with a clearly ordered lattice fringe of the BiIn phase having a (1 1 1) plane and 0.2852 nm distance. To identify the crystal structures of the composite solder, SAED patterns were obtained for each specific area, as shown in Fig. 2d–f. The SAED pattern of the specific area marked with an arrow in Fig. 2a presents the amorphous structure of the MWCNTs, as shown in Fig. 2d. The existence of various IMCs of the solder was also confirmed by the crystalline structure in the SAED pattern of area B. From this pattern, it can be seen that the phase consisted of various IMCs, the presence of which was also confirmed by the (2 0 0), (2 2 0), and (4 2 0) planes of In_3_Sn, the (1 1 0) and (1 1 1) planes of In0.2Sn0.8, and the (3 1 1) plane of BiIn, as shown in Fig. 2e and f. In particular, the EDS mapping results for the elements (the analysis is shown in Figure S8) show that the various IMCs in the composite solder were distributed separately along the interfacial boundary. As a result, the different morphology and separate distribution of all elements of the composite solder demonstrate the heterogeneity of the two different phases of the MWCNT reinforcement and the solder matrix.

**Figure 3:**
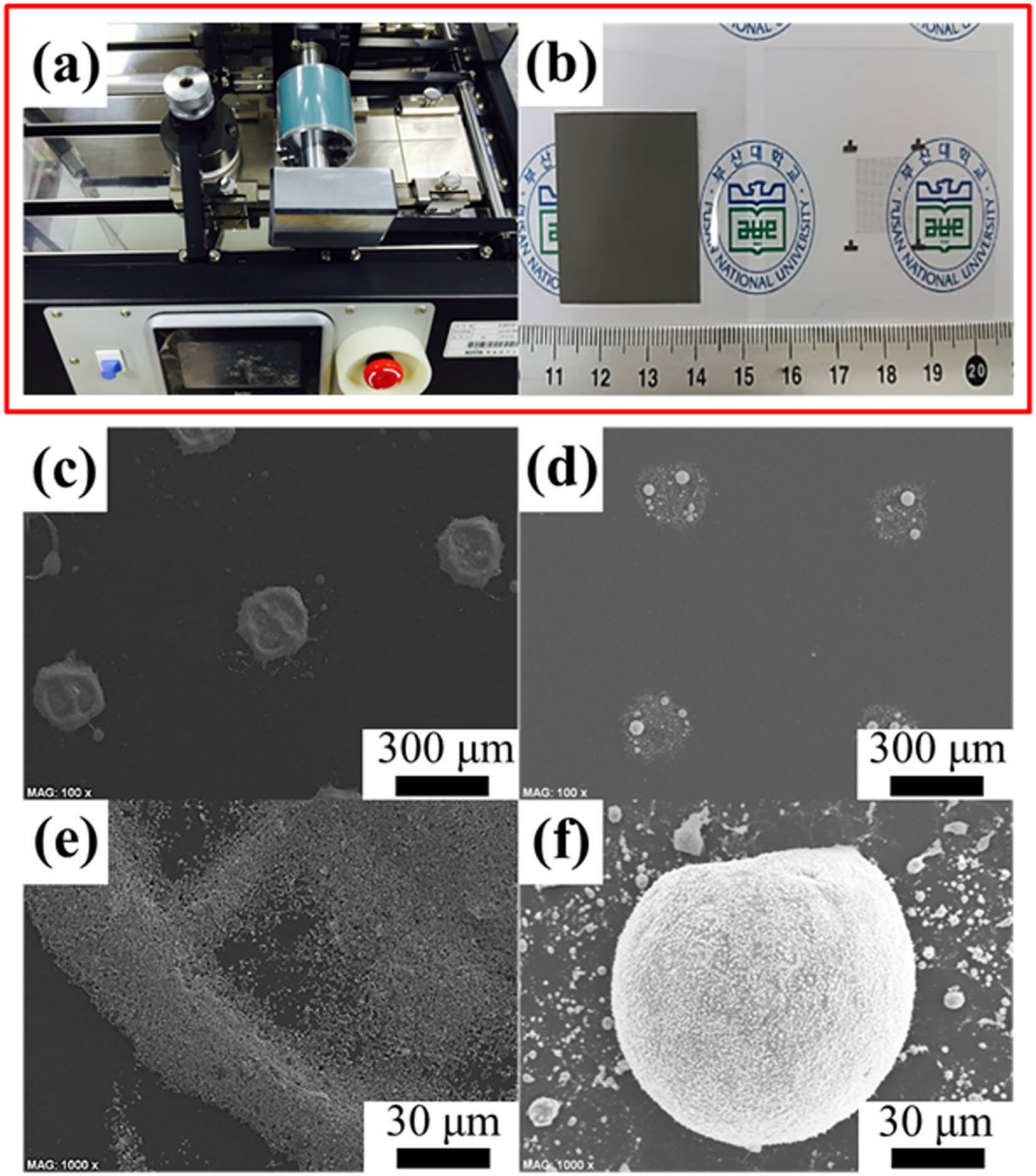
In–Sn–Bi nanoparticles are patterned on the PET substrate using a roll-to-plate printer (**a** and **b**) and reflowed at 110 °C. Two types of solder bumps printed patterns: (**b**) pad type on the left-hand side and dot and wedge type on the right-hand side. Feasibility test of In–Sn–Bi nanoparticles before (**c** and **e**) and after (**d** and **f**) reflow. The conventional solder bumps have low wettability on the PET substrate and broadly dispersed side bumps near the main core bump due to relatively low diffusivity among the solder nanoparticles.

The conventional solder bumps used for microchip interconnection were prepared by a roll-to-plate printer, as shown in Fig. 3a. In–Sn–Bi nanoparticles with a bump size of 260 µm and a pitch distance of 760 µm were attached to the flexible PET substrate using the printing process, as shown in Fig. 3b and c. After this, the solder bump arrays considerably diminished with a bump size of 210 µm but kept the same pitch distance of 760 µm (Fig. 3d). In addition, there was the morphological modification of the solder bumps. To evaluate the microstructure of the solder bumps, a higher magnification image (Fig. 3f) was examined to reveal that the solder bumps exhibited traces of the completed reflow process. A distinctive surface formed during the cooling shrinkage, and the undiffused solder nanoparticles remained on the adjacent of the center solder bump, conjugated more toward the center upon reflow soldering^2^. Meanwhile, as expected, most microstructures of the conventional solder bumps had protuberant surfaces due to the surface oxide layers of the solder nanoparticles^2^. Furthermore, the reflow status of the conventional solder bumps demonstrated poor wettability due to the heterojunction state (metal‐organic interfaces) between the metallic solder bumps and organic polymer substrates^2^. Generally, conventional Cu pads are used in practical soldering applications because they provide good wettability in reflowed solder bumps, forming specific IMCs between the solder bumps and Cu substrates such as Cu_6_Sn_5_ and Cu_3_Sn^20^. Thus, the PET substrate is typically coated with conductive metals or ceramics such as copper, nickel, cobalt, platinum, or indium tin oxide to enhance the wettability of the solder bumps^2,3,21^. However, the actual use and practical applications of the surface-modified PET substrate were omitted in our present study because the focus here was to enhance the solderability and reflowability of the solder pastes, and the flexibility and adhesion stability of the solder bumps on the PET substrate. Overall, the conventional solder bumps have poor wettability on the PET substrate.

**Figure 4:**
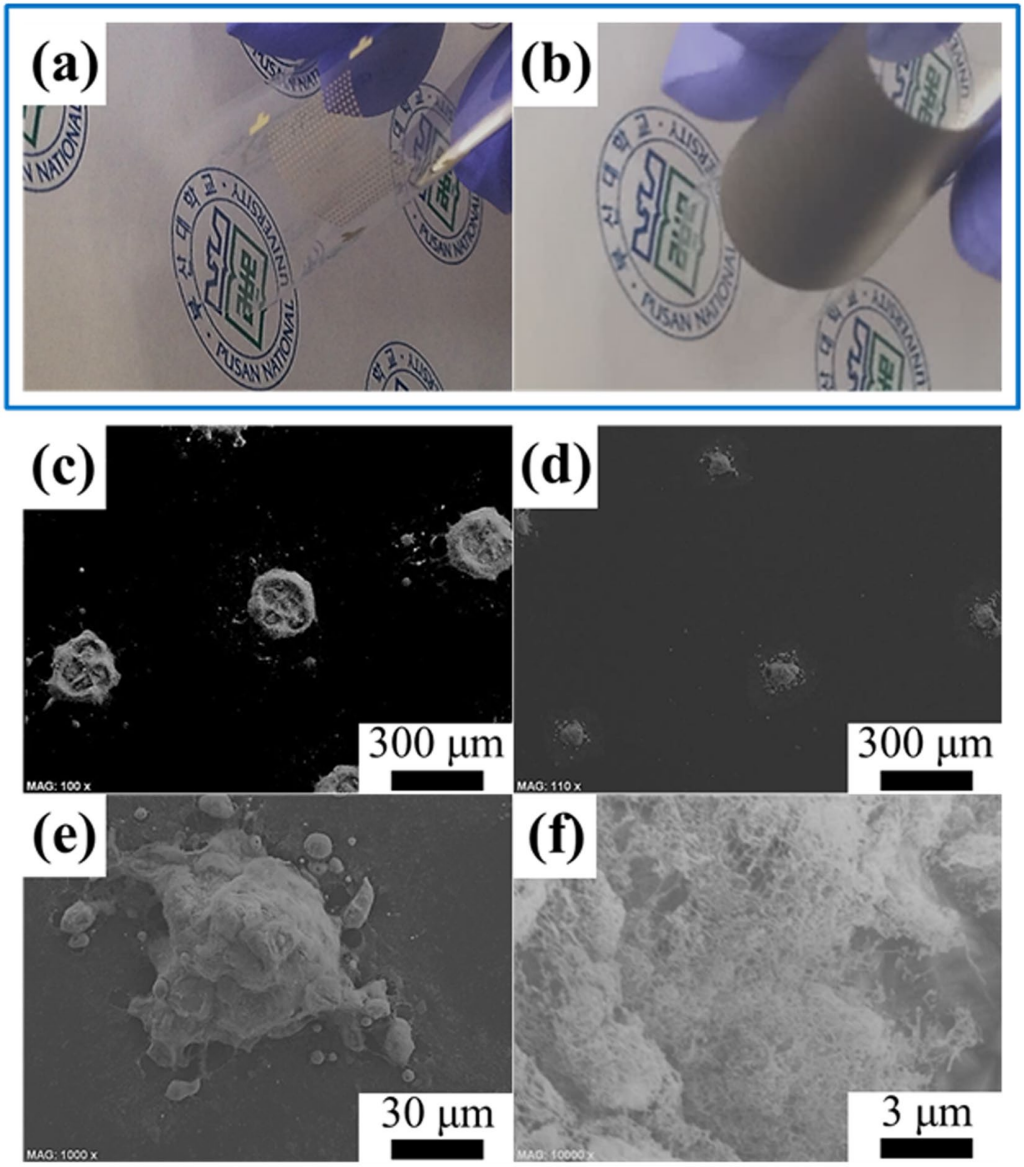
0.6MWCNT/In–Sn–Bi composite solder bumps on the PET substrate possess high flexibility despite being reflowed at 110 °C regardless of the printed pattern type of the solder bumps: dot and wedge type (**a**) and pad type (**b**). Feasibility test of 0.6MWCNT/In–Sn–Bi composite nanostructures before (**c**) and after (**d**–**f**) reflow. The composite solder bumps had high wettability, even on the PET substrate, and much fewer side bumps near the main core bump due to the improved reflowability of the In–Sn–Bi nanoparticles along the MWCNT arrays with high thermal conductivity.

The MWCNT/In–Sn–Bi composite solder bumps on the PET substrate possessed high flexibility (Fig. 4a and b). Figure 4c displays In–Sn–Bi composite solder pastes reinforced with 0.6 wt% MWCNTs patterned on the same PET substrate before the reflow process. The composite solder pastes were successfully attached to a flexible PET substrate without creating links between neighboring solder bumps before being reflowed. In addition, the shape uniformity of the solder pastes was controlled by maintaining the same pitch distance of 760 µm. Figure 4d and e shows low magnification SEM images of the consolidated solder bumps, which completely reflowed at the low temperature of 110 °C with the help of MWCNTs with a high thermal conductivity of 3000 W/(m·K)^16^. Because the thermal energy is rapidly transferred along the MWCNT arrays to the solder materials, and the solder nanoparticles have a low melting point of 98.2 °C, the reflow at a low temperature of 110 °C is sufficient to completely melt all of the composite solder nanostructures. After being reflowed, the morphology of the composite solder bumps completely changed from their agglomerated graft shapes to dome shapes. Figure 4f shows an enlarged SEM image of the composite solder bumps, depicting the interface between the MWCNT region and the solder region after the reflow. That is to say, the upper phase consisted of a lateral network structure of MWCNT arrays, while the bottom phase comprised metallic solder bumps on the PET substrate. This surface modification of the composite solder bumps is mainly ascribed to the great difference in density between the MWCNT (2.1 g/cm^3^) and solder (7.2 g/cm^3^), and the subsidiary high wettability of the composite solder with MWCNT arrays having a high thermal conductivity of 3000 W/(m·K)^16^. Consequently, the composite solder bumps had much better wettability even on the PET substrate due to the network structure of the MWCNTs that were formed after reflowing; subsequently, the solder bumps became more resistant to bending and twisting after being reflowed at 110 °C.

**Figure 5:**
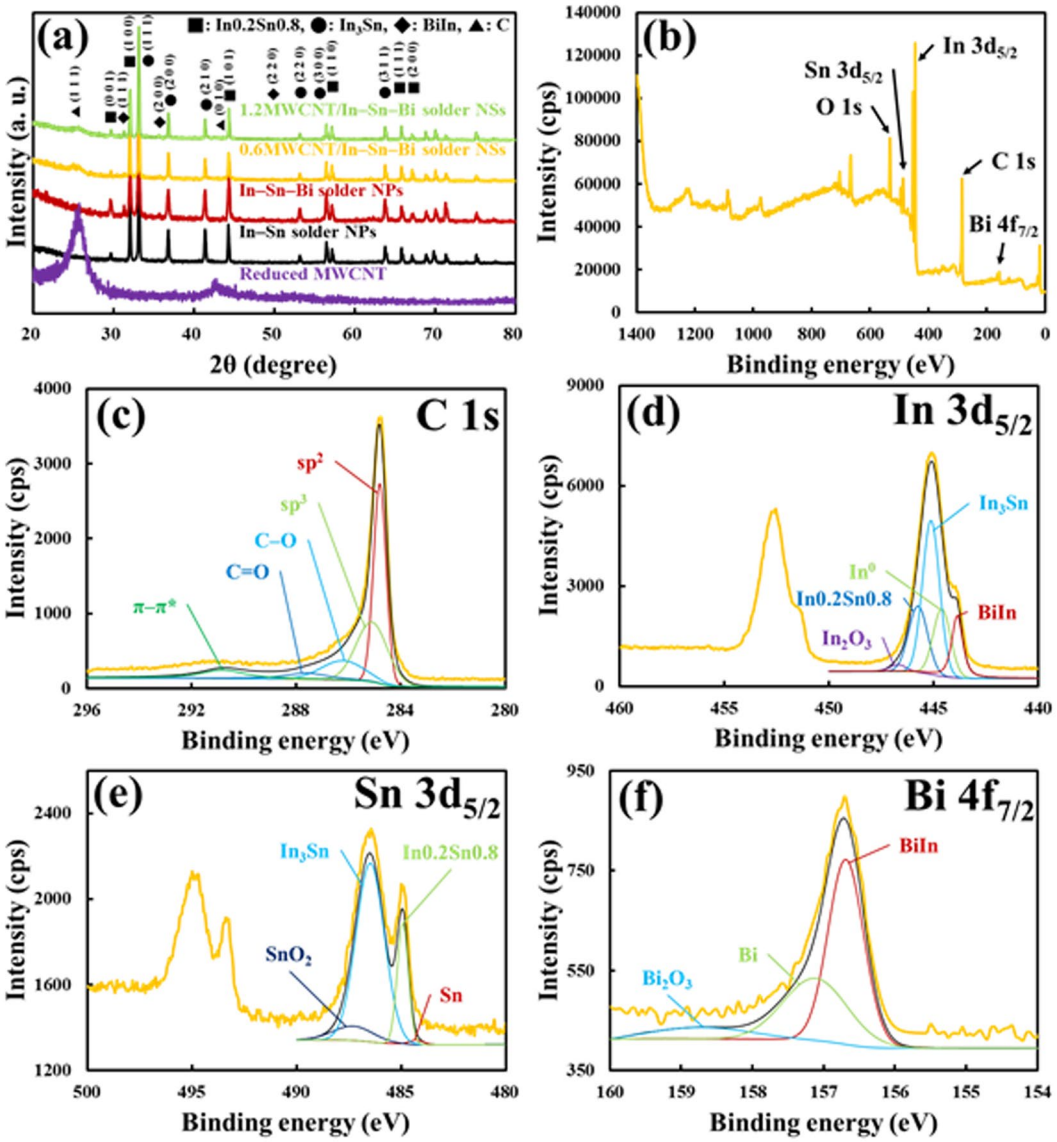
(**a**) XRD patterns of reduced MWCNTs, In–Sn and In–Sn–Bi nanoparticles, and 0.6MWCNT/In–Sn–Bi and 1.2MWCNT/In–Sn–Bi composite nanostructures. (**b**–**f**) XPS results of 0.6MWCNT/In–Sn–Bi composite nanostructures for a survey scan (**b**), C (**c**), In (**d**), Sn (**e**), and Bi (**f**). For the composite solder nanostructures, the presence of MWCNTs is ambiguous in the XRD analysis but clear in the XPS analysis, which also revealed no damage on the surface of the carbon nanotubes. The electrical and thermo-mechanical properties of the composite solder were improved.

As shown in Fig. 5a, the XRD patterns of the MWCNT/In–Sn–Bi composite nanostructures illustrate the presence of MWCNTs (JCPDS #752078) and intermetallic In_3_Sn (JCPDS #070345), In0.2Sn0.8 (JCPDS #481547), and BiIn (JCPDS #850343) compounds. The remainder of the MWCNTs was determined from the ambiguous peaks at 26.61°, 43.46°, and 46.32°. However, the formation of metal carbides, which indicate a diffusive reaction between the MWCNT arrays and the solder constituents, did not appear in the XRD patterns. The formation of various IMCs, including In_3_Sn, In0.2Sn0.8, and BiIn, was clearly indicated by their sharp peaks. The presence of In_3_Sn was identified by considering three distinguishable peaks at 2θ = 33.18°, 36.84°, and 41.42°, corresponding to the crystal planes of (1 1 1), (2 0 0), and (2 1 0), respectively, in the tetragonal crystal structure. Moreover, the diffraction peaks at 31.34°, 35.74°, and 51.44° were identified as (1 1 1), (2 0 0), and (2 2 0), which were assigned to BiIn, verifying interactions between Bi and In. However, the intensities of these peaks were relatively lower than those of In0.2Sn0.8, which had peaks at 32.06°, 44.32°, and 57.18°. The lack of BiIn in the XRD pattern probably results from the fact that the concentration of Bi was as low as 5.0 wt%, causing it to have a low intensity peak in the XRD pattern. These results also indicated that In had much higher solubility with the abundant Sn than it did with the less abundant Bi^2,11^. In any case, there was a complete lack of carbides, thus the MWCNTs were not damaged^19^. More details are presented in the XPS analysis. An XPS survey scan (Fig. 5b) was carried out to determine the elemental composition and chemical state of the composite solder nanostructures (the Gaussian–Lorentzian function and Shirley background subtraction were used for XPS spectra deconvolution). First, in order to check for the presence of carbides, we show five peaks in the C 1 s band of the MWCNTs with binding energies at 284.8, 285.2, 286.3, 287.8, and 290.8 eV assigned to C=C (sp^2^), C–C (sp^3^), C–O, C=O, and π–π*, respectively^22^. However, any specific peaks indicating metal carbides were not detected in the C 1 s band of the sample, which indicates that the MWCNTs retained the functionalities and characteristics of the composite solder related to their electrical and thermo-mechanical properties^19^. Second, the In 3p_5/2_ band in the high-resolution XPS scan was acquired at a binding energy of 445.1 eV, and after fitting, mainly consists of (1) an In_3_Sn peak at 445.1 eV and (2) an In0.2Sn0.8 peak at 445.6 eV; three minor peaks are also evident at (3) 443.9 eV, (4) 444.6 eV, and (5) 446.7 eV corresponding to BiIn, In°, and In_2_O_3_, respectively. Third, there are the two broad bands of Sn 3d_5/2_ and 3d_3/2_ at binding energies of 486.4 and 495.1 eV, respectively. In particular, there are fitted peaks at 486.4 and 485.0 eV, which correspond to the presence of In_3_Sn and In0.2Sn0.8. In the broad doublet band, the peaks of Sn^0^ and SnO_2_ are also clearly identified at 484.3 and 487.1 eV, respectively. The Bi 4f_7/2_ band in the high-resolution XPS scan also shows a binding energy of 156.7 eV and contains three different peaks with the peak at 157.1 eV being attributed to the pure metallic Bi^0^. Next, the presence of the various IMCs and the bismuth oxide phase is also revealed by deconvolution peaks for each one. Notably, the BiIn IMC on the surface of the composite solder nanostructures was identified as a major peak at 156.7 eV. The presence of bismuth oxide was identified by a peak at a binding energy of 158.8 eV, and subsequently confirmed as Bi_2_O_3_. Finally, the O 1s spectrum of the composite solder nanostructures shows a broad doublet band with a maximum peak at 532.0 eV, which can be attributed to the surface oxidation of the solder elements.

**Figure 6:**
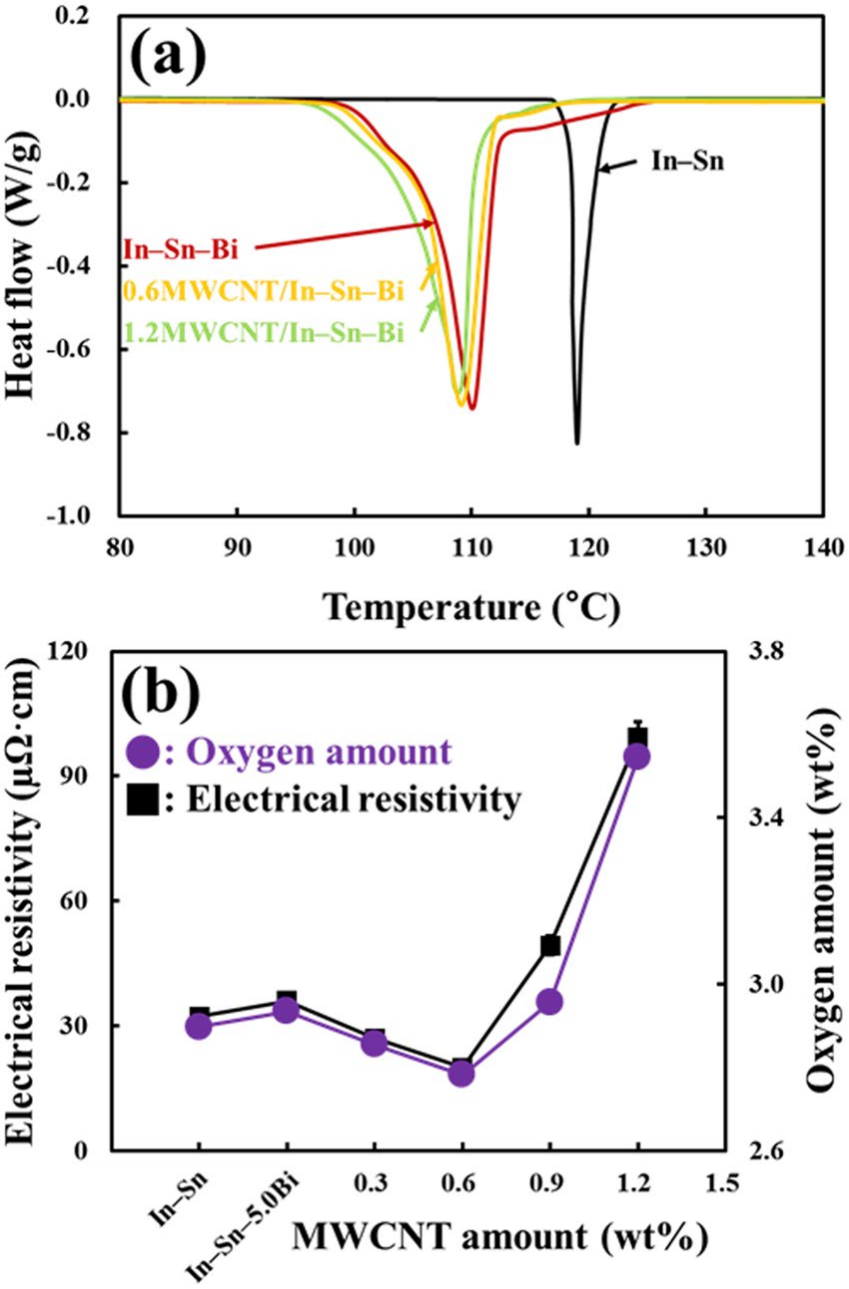
(**a**) DSC profiles of In–Sn and In–Sn–Bi nanoparticles, and 0.6MWCNT/In–Sn–Bi and 1.2MWCNT/In–Sn–Bi composite nanostructures. (**b**) Oxygen amount measurements and electrical resistivity values of the nanoparticles (or alloys) and nanostructures (or composites) with regard to chemical composition and MWCNT amount. The reinforced MWCNTs influenced the electrical properties of the composite nanostructures (or composites) rather than their thermal properties, unlike for conventional nanoparticles (or alloys).

The thermal properties of the solder are crucial factors that have to be taken into account when designing the solder reflow process. As shown in Fig. 6a, the heating processes of four different solder nanostructures were quantified using differential scanning calorimetry (DSC) analysis to determine the reflowability of each solder. First, sharp endothermic peaks emerged upon heating at 117.5 °C and 98.2 °C for the In–Sn and In–Sn–Bi nanoparticles, respectively. Second, the melting range of the In–Sn solder was as little as 4.7 °C during the DSC scan due to the presence of In_3_Sn and In0.2Sn0.8 IMCs formed between the two elements In and Sn. Third, the employment of 5.0 wt% Bi in the In–Sn alloy system increased the melting range by 15.0 °C. This increase was caused by the additional presence of BiIn IMC embedded in the binary alloy system^2,11^. Thus, the influence of the IMCs increased the melting range and directly deteriorated the reflowability of the solder^2,11^. Fourth, the melting range of the MWCNT/In–Sn–Bi composite nanostructures remained the same despite the addition of a small amount (0.6 wt%) of MWCNTs (the composition of the other solder constituents remained the same). Namely, there was no significant shift in the melting point of the MWCNT/In–Sn–Bi composite nanostructures compared to that of the In–Sn–Bi reference nanoparticles^23,24^. This can be attributed to the chemical inertness of the MWCNTs, which is suitable to obtain enough reinforcement effects without sacrificing other properties. However, the employment of greater amounts of MWCNTs (1.2 wt%) in the solder tends to be associated with a very slight decline in the melting temperature of the composite solder. This is because such a high amount of MWCNTs with high thermal conductivity of 3000 W/(m·K) in the solder plays the role of a thermal conductor, thus promotes more rapid liquidification to a small degree and a lower melting point of 97.7 °C^16,23,24^. In addition, the surface oxidation of the nanoparticles influenced the melting range and thermal reflowability of the composite solder^2^. As a result, the melting temperature negligibly or very slightly decreased as the content of MWCNTs in the In–Sn–Bi solder increased. However, the melting range was 14.8 °C for the MWCNT/In–Sn–Bi composite nanostructures, which was almost the same as that of the In–Sn–Bi nanoparticles with 15.0 °C.

Because different concentrations (0.0–1.2 wt%) of MWCNTs were added, the oxygen amounts of the composite solder nanostructures increased from 2.898 to 3.546 wt%, as shown in Fig. 6b. The high amounts of oxygen in the In–Sn conventional and In–Sn–Bi reference nanoparticles were due to surface oxidation caused by the exposure to air during the measurement process^2,25^. Although carboxyl and hydroxyl groups incorporated on the outer wall of the MWCNTs were reduced after being exposed to the strong reducing agent (lithium boron hydride), some oxide groups still remained on the surface layers of the MWCNT arrays^26^. However, the decreased oxidation of the composite solder nanostructures was due to the addition of a small concentration of reduced MWCNTs (less than 0.6 wt%) which contained a low intrinsic amount of oxygen (2.013 wt%). Thereafter, the composite solder nanostructures (more than 0.6 wt% MWCNTs) had an unexpectedly high amount of oxygen, probably due to air encapsulated in the aggregated space between their complicated graft and/or necklace structures in proportion to the amount of MWCNTs added to the solder. Herein, we assumed that the unreduced oxide groups remaining on the surface of the MWCNTs also affected the amount of oxygen in the composite solder nanostructures.

Figure 6b shows the sudden increase to 36.0 ± 0.7 µΩ·cm of the In–Sn–Bi solder compared to the electrical resistivity of the eutectic In–Sn solder (32.3 ± 0.7 µΩ·cm) caused by the addition of 5.0 wt% Bi with high electrical resistivity (theoretical value) of 129.0 µΩ·cm^27^. In fact, the electrical resistivity of both the In–Sn and In–Sn–Bi solder alloys fabricated by the reflow process (heat treatment) of their nanoparticles was significantly higher compared to the as-casted In–Sn solder alloy with low electrical resistivity of 10–15 µΩ·cm as a reference^28^. This was because the solder alloys fabricated from their nanoparticles were significantly oxidized on their outer surfaces despite the use of a strong reducing agent (lithium boron hydride), as shown in Fig. 1b. That is to say, the surface oxidation of the nanoparticles negatively influenced the electrical performance of the solder alloys quite considerably. As a result, the electrical resistivity of the In–Sn–Bi solder was higher than that of the In–Sn solder due to the addition of 5 wt% Bi with intrinsically high electrical resistivity, and the overall solder alloys fabricated by the reflow of their nanoparticles had comparatively much higher electrical resistivity values than the same compositions in the as-cast solder alloys. The electrical resistivity of the MWCNT/In–Sn–Bi composite solder was also measured using a four point probe, as shown in Fig. 6b. The measured electrical resistivity value of 19.9 ± 1.0 µΩ·cm for the In–Sn–Bi composite solder reinforced with 0.6 wt% MWCNTs was compared to the value for the In–Sn–Bi reference solder, which had electrical resistivity of 36.0 ± 0.7 µΩ·cm. Zhang *et al*. previously reported a decrease in the electrical resistivity of Sn–Bi composite solder embedded with MWCNTs, and other studies have also reported the decreased electrical resistivity of Sn–Ag–Cu solder with Ni-coated MWCNTs^16,29,30^. In practice, the electrical resistivity of the composite solder decreased by a significant value after reinforcement with up to 0.6 wt% MWCNTs. Initially, the reduced electrical resistivity was ascribed to the MWCNT reinforcement being well dispersed along the interfacial boundary of the solder matrix, after which the network arrangement of the MWCNTs led to rapid electron mobility in the composite solder. Furthermore, the intensive bonds between the MWCNT arrays and the solder constituents imparted an improvement in the electron flow channel, thus led to a decrease in the electrical resistivity of the composite solder. Because it is known that MWCNT reinforcement of the solder constituents can impart more effective electron transport, the composite solder directly attained a favorable increase in electrical conductivity^16^. Overall, the reduced electrical resistivity was ascribed to the optimum amount (0.6 wt%) of MWCNT reinforcement with high electrical performance, which thereafter was well dispersed along the grain boundaries of the solder matrix; thus, the network arrangement of the MWCNT arrays led to rapid electron mobility in the composite solder. However, reinforcement caused by greater amounts (>0.6 wt%) of MWCNTs in the composite solder did not decrease electrical resistivity; on the contrary, the results showed that electrical resistivity actually increased when more MWCNTs were added, the cause of which could be the existence of an electron transfer barrier between the MWCNT arrays and the solder constituents in the composite solder as a result of the following phenomena. First, the Schottky barrier effect, related to the electron transfer barrier, was induced between the low electrical resistivity of the carbon nanotubes and the relatively high electrical resistivity of the solder constituents^18^. In fact, it is already known that the strong interaction (high wettability) of metal atoms on the surface of carbon nanotubes can improve the electrical performance of a composite by lowering the Schottky barrier at the interface between the carbon nanotubes and the metal atoms^31–33^. In other words, strong mechanical contact between the carbon nanotubes and the metal atoms ensures minimization of the Schottky barrier at the electrical connection between them^31–33^. Following this, Cha *et al*. reported that the band gap change related to the Schottky barrier of a Ti/CNT composite was measured and compared to theoretical calculations, which revealed that the poor wetting behavior of Ti atoms on the surface of carbon nanotubes led to disruption of the electronic structure in the composite^33^. On the other hand, the effect of the high wettability of the Ti atoms on the carbon nanotubes was to lower the Schottky barrier, thus the overall electrical performance of the composite was significantly increased^33^. Based on this, we assume that the ultimate connection between the CNT arrays and the solder constituents resulted in the rapid electron flow channel by lowering the Schottky barrier and so led to a significant improvement in the electrical properties of the composite solder. In practice, the 0.6 wt% MWCNT-reinforced composite solder had the lowest electrical resistivity, as shown in Fig. 6b, due to the high wettability at the interface between the MWCNT arrays and the solder constituents. Second, despite the application of functionalized MWCNTs and the use of a strong reducing agent (lithium boron hydride) with the solder precursor ions, weak and unreacted bonds still existed between the solder constituents and MWCNT arrays^34,35^. Third, the composite solder formed micro voids and cracks when it did not completely fill in the regions between the entangled MWCNTs during the heat treatment^36,37^. These mechanical defects caused the electron-scattering phenomenon that hindered rapid electron transfer; hence, the electrical resistivity considerably increased^18^. More specifically, when the optimum amount of MWCNT arrays with high electrical properties were randomly distributed along the grain boundaries of the solder matrix, the electrons moved more rapidly along the MWCNT reinforcement presented therein, thereby enabling the composite solder to form networks that are more conductive at an optimum concentration, which decreased the electrical resistivity. In fact, a comparison between the microstructural observations in Figure S2 and the electrical conductivity measurements in Fig. 6b of the conventional solder and the composite solders manifested the afore-mentioned results by which the electrical resistivity changes of the composite solder according to MWCNT content was highly dependent on its mechanical defects. In particular, when the solder constituents were not sufficient to fill the 1.2 wt% MWNCT arrays, there were more abundant mechanical defects in the composite solder; thus, the electrical resistivity markedly increased to 99.4 ± 3.6 µΩ·cm. On the other hand, when the threshold concentration (0.6 wt%) of the MWCNT arrays with high electrical properties reinforced the solder matrix, the composite solder had less mechanical defects and thus the lowest electrical resistivity. Overall, the In–Sn–Bi composite solder reinforced with 0.6 wt% MWCNTs had the best performance from the perspective of electrical conductivity.

**Figure 7:**
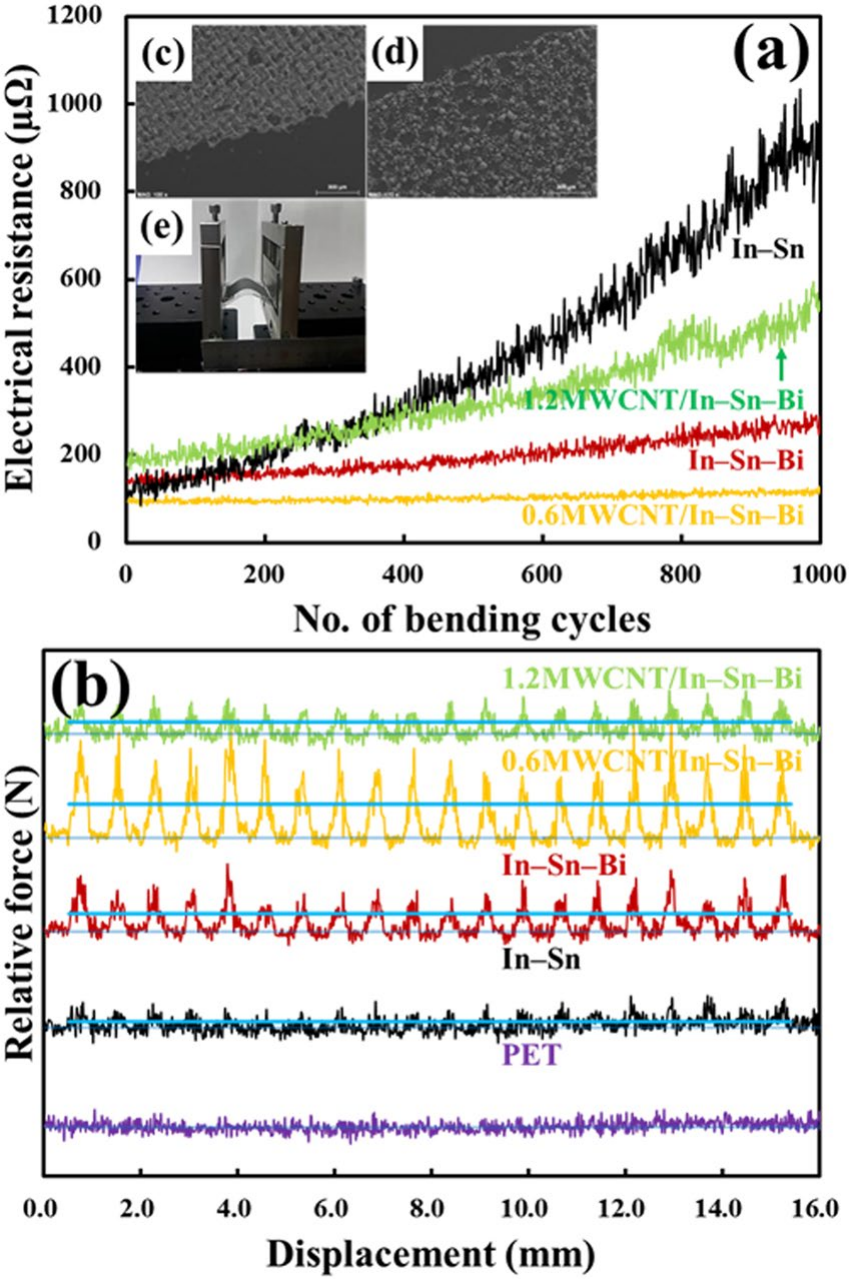
(**a**) Bending test and (**b**) scratch test on solder bumps of In–Sn, In–Sn–Bi, 0.6MWCNT/In–Sn–Bi composite, and 1.2MWCNT/In–Sn–Bi composite on the flexible PET substrate after reflow at 110 °C. The straight, opaque blue lines in (**b**) are the baselines of each scratching test, and the difference between the above line and the baseline indicates the degree of the average adhesion strength (shear force loaded) of the solder bumps on the PET substrate. Of the insets, (**c**) presents the low magnification SEM image of the 0.6MWCNT/In–Sn–Bi composite solder pads, (**d**) presents the SEM image of In–Sn conventional solder pads where contact with some regions is missing due to external bending forces, and (**e**) is an image of the bending tester used. The mechanical strength (particularly the resistance against bending and shear strength) of the composite solder pads (or bumps) was significantly improved by the MWCNT reinforcement as compared to the conventional solder pads (or bumps).

Figure 7a shows plots of the changes in electrical resistance versus the bending cycles of conventional and composite solder bumps (pads). Initially, the application of bending stress produced some tearing and fracturing of the In–Sn conventional solder pads on the PET substrate, which is most likely due to the limited intrinsic bendability of the In–Sn solder pads^38^. Although Sn has an even higher fracture toughness (15–30 MPa·m^1/2^) than In (3–7 MPa·m^1/2^), two types of IMCs (In_3_Sn and In0.2Sn0.8) were produced with much lower fracture toughness values due to their intrinsic brittleness^39,40^. Furthermore, the In–Sn nanoparticles did not reflow properly at 110 °C due to their relatively high melting point (117.5 °C), which induced sintering only and less melting in the solder pads. The same bending test was also conducted with the In–Sn–Bi solder pads, which the reference solder pads tolerated relatively well, with repeated bending during the same number of cycles (1000) and under the identical condition of compressive/tensile stress–strain. Consequently, the electrical resistance gradually increased (R/R_0_ = 2.4) because the In–Sn–Bi solder has a melting point of 98.2 °C and reflowed well during the heat treatment at 110 °C. Subsequently, bending the composite solder pads produced an almost negligible change in the electrical resistance for the In–Sn–Bi composite solder pads reinforced with 0.6 wt% MWCNTs on the PET substrate. Specifically, the MWCNT/In–Sn–Bi composite solder pads adhered strongly to the underlying PET substrate and became robust against damage due to bending, twisting, and flexing. Thus, the composite solder pads passed the bending test without a significant change in electrical resistance or loss of pads attached to the PET substrate, and remained almost constant when films were plastically deformed by bending. Furthermore, the solder residues even remained constant after vigorous bending several times with two fingers. As a result, the electrical resistance of the MWCNT/In–Sn–Bi composite solder pads on the PET substrate remained relatively constant throughout the testing cycles after repetitive bending and relieving cycles on the same area. Even after 1000 cycles, there was essentially no change in resistance (R/R_0_ = 1.2). For more details, refer to inset (c) in Fig. 7a, which shows a micrograph of the MWCNT/In–Sn–Bi composite solder pads after exposure to 1000 bending cycles. The uniform spread of the conductive residues occurred without any missing parts of solder pads, while the In–Sn conventional solder pads on the PET substrate exhibited poor bending resistance, which was evident from micrographs of a large area of missing reflowed solder pads, as shown in inset (d) in Fig. 7a, after the same 1000 bending cycles. The solder pads were loaded on a bending tester, as shown in inset (e) of Fig. 7a. However, the flexibility of In–Sn–Bi composite solder pads reinforced with 1.2 wt% MWCNTs on the PET substrate did not extend for many bending cycles. Instead, the electrical resistance of MWCNT/In–Sn–Bi composite solder pads considerably increased (>3-fold: R/R_0_ = 3.1). This was because addition of too much MWCNTs (1.2 wt%) embedded in the solder pads on the PET substrate increased the potential for mechanical defects of the composite solder pads caused by the chemical inertness of the MWCNTs. It is generally known that electrical performance of a MWCNT-reinforced composite depends strongly on the mechanical defects in the matrix, especially according to the dispersion, length, crystallinity, and degree of alignment of the carbon nanotube reinforcement as well as the binding characteristics between the carbon nanotubes and the matrix constituents^16,36,41–45^. Meanwhile, the strong interaction between the MWCNT arrays and the matrix constituents is a balance between the optimum amount of MWCNT reinforcement to produce high electrical performance and mechanical defects in the matrix caused by the unreacted surplus MWCNT arrays^16,36,41–45^. Following this, strongly anchoring the matrix on the surface of the MWCNT reinforcement has been the key issue in improving the electrical performance of the composite^16,36,41–45^. Otherwise, poorly facilitating the mechanical strength of the composite leads to degraded electrical performance^16,36,41–45^. In practice, the electrical resistance of the composite solder remained almost constant from 105.1 to 110.3 µΩ during the 1000 bending cycles when it contained the threshold amount (0.6 wt%) of MWCNTs optimized for strong interactions between the MWCNT arrays and the solder constituents, as shown in Figure S2f. However, when the composite solder included MWCNT arrays at an excessively high amount (1.2 wt%), it contained many mechanical defects, as shown in Figure S2h. Thus, the electrical resistance significantly increased from 175.8 to 551.9 µΩ during the same bending cycles, as shown in Fig. 7a. As a result, the addition of too much MWCNTs (1.2 wt%) to the solder nanoparticles badly deteriorated the flexibility of the composite solder pads because these poor bonds formed pores or cracks at the grain boundaries of the composite solder; hence, the electrical resistance rapidly increased with the addition of more MWCNTs.

A scratch test was conducted to measure the adhesion strength of the composite solder bumps, the results of which are shown in Fig. 7b. There are 20 dot arrays with a pitch distance of 760 µm in the pattern, and the bonding strength of each solder bump differed at the same applied shear force of 10.0 N, based on the peak intensity change^2^. A scratch test for the raw PET substrate without any solder bumps was conducted as a reference. Since there were no solder bumps, the relatively high fluctuation of the peaks constituted the signal-to-noise ratio. For the scratch test on the In–Sn solder bumps, there was very poor adhesion stability because the binary In–Sn nanoparticles had a high melting point of 117.5 °C, and so the solder bumps did not reflow at 110 °C. Comparatively, the In–Sn–Bi solder bumps had higher adhesion strength (average shear force of 0.5 N) due to their lower melting point (98.2 °C) and the intrinsic ductility of In and Sn, although the amount of IMCs increased with regard to the Bi interactions between In and Sn that resulted in increased brittleness in the solder bumps^2^. Subsequently, the In–Sn–Bi composite solder bumps reinforced with 0.6 wt% MWCNTs had the highest adhesion strength (average shear force of 0.9 N) because they were tightly tied to the lateral network structure of the MWCNT arrays on the solder materials and had the highest detachment resistance to the external shear force, which thus led to the highest adhesion strength in the solder bumps. The high reflowability of the composite solder nanostructures also imparted high adhesion strength to the solder bumps on the PET substrate. The solder nanoparticles had an intrinsic low melting point of 98.2 °C, and so were diffused well along the MWCNT arrays at the sufficiently high reflow temperature of 110 °C. However, adding too much reinforcement (>1.2 wt% MWCNTs) to the solder nanoparticles resulted in decreased adhesion strength (average shear force of 0.2 N). As was similarly discussed above in the bending test section, this is because the high chemical inertness of the MWCNTs induced mechanical defects, such as pores and cracks, in the composite solder bumps, although the functionalized MWCNTs were used for the reinforcement^16,36^. As a result, the composite solder bumps with the optimal amount (0.6 wt%) of reinforced MWCNTs had high maximum adhesion strength due to their improved wettability and diffusivity, without the sacrifice of any other properties.

## Conclusions

Novel MWCNT/In–Sn–Bi composite nanostructures of incorporated MWCNT arrays with high electrical and thermal conductivity and reinforced In–Sn–Bi nanoparticles with a low melting temperature of 98.2 °C were synthesized using a chemical reduction method for attachment to a flexible PET substrate. In particular, the composite solder with 0.6 wt% MWCNTs achieved low electrical resistivity (19.9 ± 1.0 µΩ·cm) because MWCNTs behaved like an “express tunnel” for rapid electron transfer in contrast with the “turbulent traffic” of the In–Sn–Bi reference solder with much higher electrical resistivity (36.0 ± 0.7 µΩ·cm). Such high electrical performance was attributed to a network structure optimized for rapid electron transport, which resulted from maximizing the composite solder’s ability to transport electrons. Afterwards, the composite solder nanostructures were reflowed at a low temperature of 110 °C, during which the PET substrate was not damaged. Next, we showed that tight adherence to the plastic substrate with high flexibility (17.7% slope of resistance at 1,000 cycles of operation in a bending test) and strong adhesion stability (0.9 N loaded force in a scratch test) was achieved by the composite solder bumps. Therefore, the novel MWCNT/In–Sn–Bi composite nanostructures can provide significant advancement of the interconnectability of electronic components and soldering technology for use in flexible microelectronic packaging industries.

## Materials and Methods

A small amount (0.3 g) of MWCNTs (≥98.0%, 6–13 nm diameter × 2.5–20 µm length, Sigma Aldrich, USA) were dispersed in piranha solution (a mixture of 70:30 sulfuric acid and hydrogen peroxide) in a round bottom flask equipped with a condenser. The functionalized MWCNTs were washed until neutral pH was attained, then the precursor was dried in a vacuum. Subsequently, 1.1 mg of functionalized MWCNTs were mixed together with 9 mL of oleylamine and then sonicated for 30 min to form a stable suspension. The suspension was supplemented with 0.238 g of In(NO_3_)_3_·xH_2_O (≥99.999%, Sigma Aldrich, USA) and 0.287 g of Sn(Oct)_2_ (≥92.5%, Sigma Aldrich, USA) in the presence of 0.021 g of Bi(NO_3_)_3_·5H_2_O (≥99.999%, Sigma Aldrich, USA) as nanoparticle precursors, and 5.0 × 10^−5^ mol of 1,10-phenanthroline (≥99%, Sigma Aldrich, USA) was used as a surfactant. Next, 5.0 × 10^−3^ mol of lithium boron hydride was added as a reducing agent, then sonication was carried out for a further 30 min. The mixed solution was heated and kept at 80 °C for 2 h in argon gas, during which time the In–Sn–Bi nanoparticles became nucleated on the surface of the MWCNTs. After the chemical reduction, the MWCNT/In–Sn–Bi composite nanostructures were further purified by a centrifugal process. Using the same chemical method, each combination of In, Sn, and Bi ions (in the absence of functionalized MWCNTs) provided In–Sn and In–Sn–Bi nanoparticles, respectively. The composite solder nanostructures were blended with epoxy resin and polyethylene glycol at a 90:5:5 weight percentage ratio using a three-roll miller (EXAKT 50 I, EXAKT Technologies, Inc., USA) to prepare composite solder pastes that were then each filmed with dot array and rectangular patterns on a PET (50 mm × 50 mm) substrate, and prepared using a roll-to-plate printer (iPen Co., Ltd., Republic of Korea). The solder pastes were then reflowed at 90 °C for 1 min and 110 °C for 1 min in a glove box filled with argon gas.

To observe the effects of reinforcement of the solder matrix elements (In, Sn, and Bi) with the MWCNTs, incrementally greater amounts of MWCNTs were added while the amounts of the other solder elements remained fixed, as shown in Table 1. Each constituent of various solders was measured using a balance (EL204–IC, METTLER TOLEDO, Switzerland) with repeatability (standard deviation) of ±0.1 mg. Following this, the composite solder nanostructures were compacted at a constant axial pressure of 5000 psi into identical disc-shaped pellets, after which all solder samples were heat treated at 90 °C for 1 min and 110 °C for 1 min in argon gas. Next, the heat-treated samples were polished using diamond paste and then cleansed by sonication for 10 min after they had been fully submerged inside anhydrous ethanol. Microstructure images and energy dispersive X-ray spectroscopy (EDS) data were acquired using a scanning electron microscope (SEM, JSM–6610/LV, JEOL, Japan). The electron diffraction of the crystal orientation was analyzed using the selected area electron diffraction (SAED) unit of a field emission transmission electron microscope (TEM, Tecnai F30 S-Twin, FEI, USA). The crystalline structure of the composite solder was analyzed with an X-ray diffractometer (XRD, D/Max–2500VL/PC, Rigaku International Corporation, Japan). An X-ray photoelectron spectrometer (XPS, Quantera SXM, ULVAC–PHI, Japan) and XPSPEAK 4.1 software were used to obtain more detailed information about the composition of the compounds in the composite solder. The weight percent of the oxygen and carbon contained in the composite solder nanostructures were determined using an oxygen/nitrogen analyzer (ON–900, ELTRA GmbH, Germany) and a carbon/sulfur analyzer (CS–800, ELTRA GmbH, Germany) with 0.01 ppm sensitivity and ± 0.1 ppm accuracy for a 1.0 g sample, respectively^2,46^. A four-point probe (FPP–RS8, Dasol Engineering, South Korea) was used to measure the electrical resistivity of the cross-section of the composite solder. The electrical resistivity measurements were affected by environmental moisture, room temperature, measurement skill, human and instrument error, and so on. The thermal property of the composite solder nanostructures was measured using a differential scanning calorimeter (DSC, Q100, TA Instrument, USA). The flexibility of the composite solder bumps was determined using a custom-made bending tester. Similarly, the adhesion strength of the solder bumps was determined by a scratch test, which consisted of pulling a diamond stylus over the surface of the solder bump series under normal force. The diamond stylus has Rockwell C geometry with a 120° cone and the 200 µm radius spherical tip. The loading force used was 10.0 N, and the indenter transverse speed was 2.0 mm/min.

**Table 1:** Chemical composition, oxygen affinity, and carbon amount of In–Sn conventional nanoparticles, In–Sn–Bi reference nanoparticles, and MWCNT/In–Sn–Bi composite nanostructures.

	In (wt%)	Sn (wt%)	Bi (wt%)	MWCNT (wt%)	O (wt%)	C (wt%)
A	52.0	48.0	—	—	2.898	—
B	49.4	45.6	5.0	—	2.935	—
C	49.3	45.5	5.0	0.3	2.856	0.297
D	49.1	45.3	5.0	0.6	2.782	0.599
E	49.0	45.2	5.0	0.9	2.957	0.903
F	48.8	45.1	4.9	1.2	3.546	1.202

## Electronic supplementary material

Supplementary information
