# Supplementary material for: Improved electrical and thermo-mechanical properties of a MWCNT/In–Sn–Bi composite solder reflowing on a flexible PET substrate
Source: Sci Rep. 2017 Oct 23;7:13756. doi: 10.1038/s41598-017-14263-6 (PMC5653769; doi:10.1038/s41598-017-14263-6)
Supplement: Supplementary file 1 — Supplementary information [file 41598_2017_14263_MOESM1_ESM.doc]

**Improved electrical and thermo-mechanical properties of a MWCNT/In–Sn–Bi composite solder reflowing on a flexible PET substrate**

Sang Hoon Kim^1,2,*^, Min-soo Park^1,2^, Joon-Phil Choi^3^, and Clodualdo Aranas Jr.^3^

^1^Powder Technology Department, Korea Institute of Materials Science, Changwon 51508, Republic of Korea

^2^School of Materials Science and Engineering, Pusan National University, Busan 46241, Republic of Korea

^3^Department of Mining and Materials Engineering, McGill University, 3610 University Street, Montreal, QC, H3A 0C5, Canada

*Corresponding Author: Dr. Sang Hoon Kim: sanghooni791@naver.com

Although In–Sn solder with a eutectic composition has a low electrical resistivity of 32.3 ± 0.7 µΩ·cm, the binary nanoparticles have an inappropriately high melting point (117.5 °C) for reflowing on a flexible PET substrate because high reflow temperatures (> 110 °C) can damage the plastic substrate. Based on the metallurgy, the addition of an appropriate amount of additives can lower the melting point of the binary solder system due to movement toward a more eutectic melting temperature; for example, the addition of 5.0 wt% Bi can lower the melting point of In–Sn–Bi nanoparticles to 98.2 °C, a temperature at which the PET substrate does not decompose. However, the electrical resistivity of the ternary In–Sn–Bi solder (36.0 ± 0.7 µΩ·cm) becomes higher than that of the binary In–Sn solder (32.3 ± 0.7 µΩ·cm) due to the intrinsically high electrical resistivity of Bi (129.0 µΩ·cm) and the formation of more solid solutions (e.g. BiIn, among others) at the grain boundaries, resulting in more electron scattering. Thus, electron-active carbon nanostructures such as MWCNTs with high electrical and thermal conductivity and strong mechanical strength were introduced as one of the best types of solder reinforcement material, especially for In–Sn–Bi nanoparticles, in a solder intended to be reflowed on the PET substrate. Subsequently, after the MWCNTs were added at different concentrations, the optimized amount of MWCNTs embedded at the core of the In–Sn–Bi nanoparticles was found in terms of the resulting solder’s electrical and thermo-mechanical performance.

As shown in **Figure S2**a and c, the synthesized In–Sn and In–Sn–Bi nanoparticles exhibit severe agglomeration due to van der Waals interactions. Furthermore, the cross-sectional SEM images of each solder (**Figure S2**b and d) fabricated after heat treatment in inert gas, polished with diamond paste, and etched with acids presented grains of different sizes with various segregated solid solutions at the grain boundaries. In particular, there were In-rich phases and Sn-rich phases, as shown in **Figure S3**. Their overlap regions were represented on the surface of the binary In–Sn solder, revealing the presence of IMCs (In_3_Sn and In0.2Sn0.8), which can be seen in the very fine regular segregates. As shown in **Figure S4**, the small amount (5.0 wt%) of Bi resulted in more segregated Bi formation at the grain boundaries of the ternary In–Sn–Bi solder. Bi does not form any IMCs with Sn but does with In; hence, in the EDS mapping images of the ternary In–Sn–Bi solder, the distribution image of Bi overlaps with that of In. This indicates the additional formation of BiIn IMC, which can greatly affect the electrical and thermo-mechanical properties of the solder joints. Indeed, numerous studies indicate that excessive formation of IMCs may promote brittle failure by weakening solder joint strength, and hence affect long term solder joint reliability. As shown in **Figure S2**e and g, grafting of the In–Sn–Bi nanoparticles on the surface of the MWCNT arrays obviously occurred even though the solder nanoparticles were randomly attached and of differing sizes in the tubular structure of the MWCNTs. Furthermore, the composite solder nanostructures had broad size distribution, and the extent of agglomeration of the composite solder nanostructures was particularly dependent on the graft size from which it formed. **Figure S2**f and h show cross-sectional SEM images of each composite solder containing 0.6 and 1.2 wt% MWCNTs and having obtained the corresponding EDS mapping images (**Figure S5**). Comparison of the SEM images of the MWCNT/In–Sn–Bi composite solders with the In–Sn and In–Sn–Bi solders revealed the unique role of the MWCNTs as a reinforcement material leading to a lateral network structure at the grain boundaries after being heat-treated. Overall, this explains the high electrical and thermo-mechanical performance of the composite solder bumps.

**Figure S9** shows the morphology of the In–Sn–Bi conventional and MWCNT/In–Sn–Bi composite solder bumps on the Cu substrate after reflowing at 110 °C, while **Figure S9**a illustrates the dome shape of the In–Sn–Bi solder bumps, which was different from their spherical shape on the PET substrate (**Figure 3**d). However, the solder bumps still maintained an average pitch distance of 760 μm between adjacent solder bumps, which were successfully attached without creating links between neighboring solder bumps on the Cu substrate. **Figure S9**c depicts enlarged detail of a single solder bump more broadly dispersed with higher diffusivity and wettability than the disorderly distributed solder bumps consisting of one in the middle surrounded by others on the PET substrate (**Figure 3**d). In **Figure S9**e, we can see that the surface of the solder bumps on the Cu substrate became smoother than that on the PET substrate (**Figure 3**f). The MWCNT/In–Sn–Bi composite solder bumps (**Figure S9**b and d) were more widely dispersed after being reflowed on the Cu substrate compared to those on the PET substrate (**Figure 4**d and e). In particular, a comparison of their morphology determined that the composite solder bumps on the Cu substrate had even more increased diffusivity. They also had better reflowability because of the wettability improvement caused by the effect of the MWCNT arrays with high thermal conductivity of 3000 W/(m·K) in the upper phase on the metallic solder bumps in the bottom phase, as shown in **Figure S9**f^1, 2^. In this respect, the morphology of the composite solder bumps on the Cu substrate was similar to that of the composite solder bumps on the PET substrate in that the upper phase consisted of a lateral network structure of MWCNT arrays while the bottom phase comprised metallic solder bumps on the Cu substrate.

As shown in **Figure S10**, the cross-sectional SEM images of the In–Sn–Bi conventional solder on the Cu substrate are compared to those of the MWCNT/In–Sn–Bi composite solder. In fact, the formation of specific phases and IMCs between In–Sn and Bi–Sn solder alloys and the Cu substrate has already been reported in the literature^3, 4^. In particular, for Sn-based solders on the Cu substrate, it has become well established that the reaction (diffusion) layer is formed and consists of the intermetallic Cu_3_Sn compound at a temperature above 350 °C and the more common intermetallic Cu_6_Sn_5_ compound at a temperature below 350 °C^4, 5^. **Figure S10**a shows that the thin diffusion layer of the conventional solder is clearly observable at the solder/Cu interface. This layer was not homogeneous in thickness, and furthermore, many micro voids and cracks were present between the solder layer and the diffusion layer, as can be better seen in the high-resolution SEM image in **Figure S10**c. The average thickness of the layer was about 110 μm, and the diffusion between the solder and the substrate led to the formation of specific phases at the interfacial layer revealed by the strong acid etching that varied from 20 to 120 μm. In particular, the diffusion layer contained small-sized distinctive phases (**Figure S10**e) that were very similar to the microstructures of intermetallic Cu_11_In_9_ and Cu_6_Sn_5_ compounds reported in the literature^6, 7^. An EDS mapping analysis (**Figure S11**) of the conventional solder shows that the complicated shape of the phases at the interfacial layer consisted of a large fraction of a Cu-rich phase with small fractions of In- and Sn-rich phases. A cross-sectional image of the MWCNT/In–Sn–Bi composite solder on the Cu substrate is also presented in **Figure S10**b. Although the reinforcement of 0.6 wt% MWCNTs should impart a supplementary MWCNT layer to the composite solder in addition to the three pre-existing layers between the solder and the substrate, the MWCNT layer present in the top layer of the composite solder was mostly removed by the polishing and etching process. In addition, the presence of the diffusion layer at the solder/Cu interface was ambiguous because the MWCNT arrays suppressed the formation of new Cu–In and Cu–Sn phases (IMCs), although some traces of these new phases remained. That is to say, the thermally and chemically inert carbon nanotubes slightly interrupted the brittle IMC formation, thus there were much fewer micro voids and cracks at the boundaries between the solder and diffusion layers than in the conventional solder, as shown in **Figure S10**d and f^2, 8-10^. **Figure S12** contains an SEM image and the corresponding EDS mapping images of the diffusion layer of the composite solder on the Cu substrate, and these also proved that the MWCNT arrays suppressed the formation of the new IMCs.

**Figure S13** shows the XRD patterns of the In–Sn–Bi conventional solder and the MWCNT/In–Sn–Bi composite solder reflowed (diffused) on the Cu substrate. From the previous XRD pattern of In–Sn–Bi solder (**Figure 5**a), In_3_Sn (JCPDS #070345), In0.2Sn0.8 (JCPDS #070345), and BiIn (JCPDS #850343) IMCs were present. However, the diffusion of the solder on the Cu substrate resulted in the appearance of new Cu-containing IMCs, such as Cu_11_In_9_ (JCPDS #410883) and well-known Cu_6_Sn_5_ (JCPDS #451488)^4^. On the other hand, this resulted in a decrease of the previously formed In_3_Sn, In0.2Sn0.8, and BiIn IMCs due to the active diffusion of In, Sn, and Bi with Cu. This result was very similar to reports by Noor and Xian in which they found that Cu_11_In_9_ and Cu_6_Sn_5_ IMCs were formed at the interfacial layer of the In–Sn solder when it was being reflowed on the Cu substrate^4, 7^. In practice, the XRD analysis shows that there were many 2θ-matching peaks with relatively increased intensities for the Cu_11_In_9_ and Cu_6_Sn_5_ IMCs compared to the lowered intensity peaks of the In_3_Sn and In0.2Sn0.8 IMCs, as shown in **Figure 5**a. In addition, the phase diagrams of Cu–In and Cu–Sn point toward the formation of new intermetallic Cu_11_In_9_ and Cu_6_Sn_5_ compounds that started to form at around 305.8 °C and 415.0 °C, respectively^6, 11-13^. Meanwhile, the phase diagram of In–Sn presents the intermetallic In_3_Sn and In0.2Sn0.8 compounds, formed near 130.0 and 190.0 °C, respectively, which signifies that the Cu_11_In_9_ and Cu_6_Sn_5_ IMCs were thermally and energetically more stable than the In_3_Sn and In0.2Sn0.8 IMCs^6, 11-13^. From the XRD pattern of the composite solder, the formation of new IMCs and the decrease of the previously formed IMCs were almost identical to that of the conventional solder. Furthermore, the presence of the MWCNTs in the composite solder is still ambiguous due to their small amount (0.6 wt%) of reinforcement. Overall, we determined that both the conventional and composite solders reflowed well on the Cu substrate, even at the low temperature of 110 °C, which was proved by the formation of new Cu-containing IMCs.

**References**

1. Zhang, S. & Chen, Q. Fabrication of MWCNT incorporated Sn–Bi composite. *Composites Part B: Engineering* **58**, 275-278 (2014).

2. Billah, M. M. & Chen, Q. Thermal conductivity of Ni-coated MWCNT reinforced 70Sn–30Bi alloy. *Composites Part B: Engineering* **15**, 162-168 (2017).

3. Li, J., Mannan, S., Clode, M., Whalley, D. & Hutt, D. Interfacial reactions between molten Sn–Bi–X solders and Cu substrates for liquid solder interconnects. *Acta Materialia* **54**, 2907-2922 (2006).

4. Noor, E. E. M. *et al*. Wettability and strength of In–Bi–Sn lead-free solder alloy on copper substrate. *J. Alloys Compounds* **507**, 290-296 (2010).

5. Frear, D. R., Burchett, S. N., Morgan, H. S. & Lau, J. H. *Mechanics of solder alloy interconnects* (Springer Science & Business Media, 1994).

6. Aljilji, A., Minić, D., Manasijević, D., Živković, D. & Todorović, A. Phase equilibria and thermodynamics of the Bi–Cu–In ternary system. *Thermochimica Acta* **498**, 11-15 (2010).

7. Xian, J., Ma, Z., Belyakov, S., Ollivier, M. & Gourlay, C. Nucleation of tin on the Cu_6_Sn_5_ layer in electronic interconnections. *Acta Materialia* **123**, 404-415 (2017).

8. Sun, H., Chan, Y. & Wu, F. Effect of CNTs and Ni coated CNTs on the mechanical performance of Sn57.6Bi0.4Ag BGA solder joints. *Materials Science and Engineering: A* **656**, 249-255 (2016).

9. Dele-Afolabi, T., Hanim, M. A., Norkhairunnisa, M., Yusoff, H. & Suraya, M. Investigating the effect of isothermal aging on the morphology and shear strength of Sn–5Sb solder reinforced with carbon nanotubes. *J. Alloys Compounds* **649**, 368-374 (2015).

10. Khodabakhshi, F., Sayyadi, R. & Javid, N. S. Lead free Sn–Ag–Cu solders reinforced by Ni-coated graphene nanosheets prepared by mechanical alloying: Microstructural evolution and mechanical durability. *Materials Science and Engineering: A* (2017).

11. Fürtauer, S., Li, D., Cupid, D. & Flandorfer, H. The Cu–Sn phase diagram, Part I: new experimental results. *Intermetallics* **34**, 142-147 (2013).

12. Bahari, Z., Elgadi, M., Rivet, J. & Dugué, J. Experimental study of the ternary Ag–Cu–In phase diagram. *J. Alloys Compounds* **477**, 152-165 (2009).

13. Chu, S., Schwartz, A. J., Massalski, T. B. & Laughlin, D. E. Extrinsic paramagnetic Meissner effect in multiphase indium–tin alloys. *Appl. Phys. Lett.* **89**, 111903 (2006).


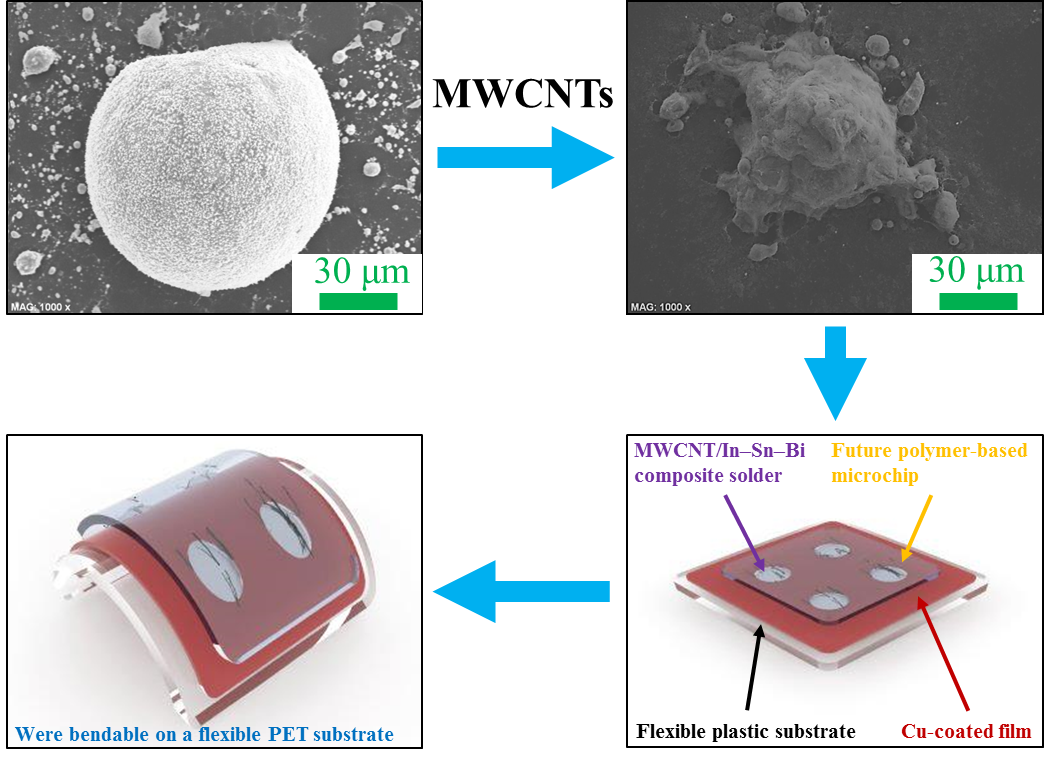


**Figure S1.** The reflow of 0.6MWCNT/In–Sn–Bi composite solder pastes on a flexible PET substrate.

**
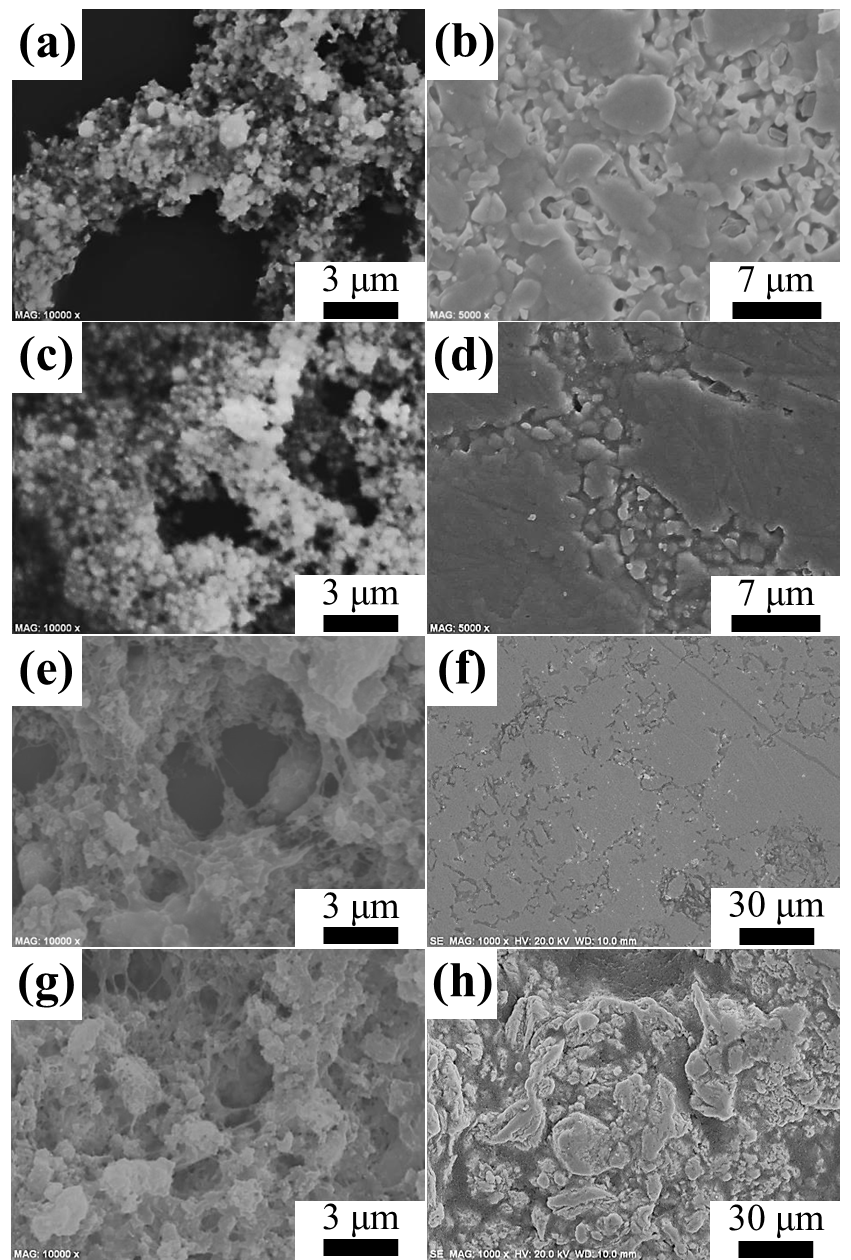
**

**Figure S2.** SEM images of In–Sn (a) and In–Sn–Bi nanoparticles (c), and 0.6MWCNT/In–Sn–Bi (e) and 1.2MWCNT/In–Sn–Bi composite nanostructures (g). Cross-sectional SEM images of In–Sn (b) and In–Sn–Bi alloys (d), and 0.6MWCNT/In–Sn–Bi (f) and 1.2MWCNT/In–Sn–Bi composites (h).


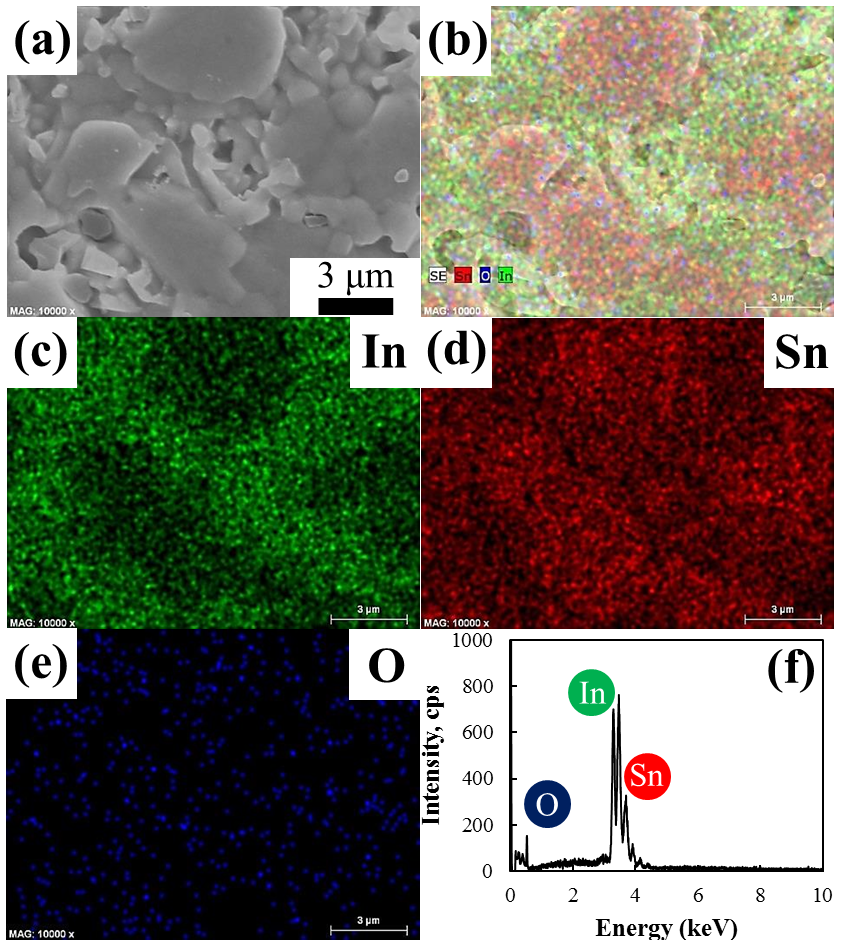


**Figure S3.** Cross-sectional SEM image (a), mapping of the analysis results (b–e), and EDS analysis of In–Sn alloy (f) with In L_α_ (3.286 keV), Sn L_α_ (3.443 keV), and O K_α_ (0.525 keV).

**
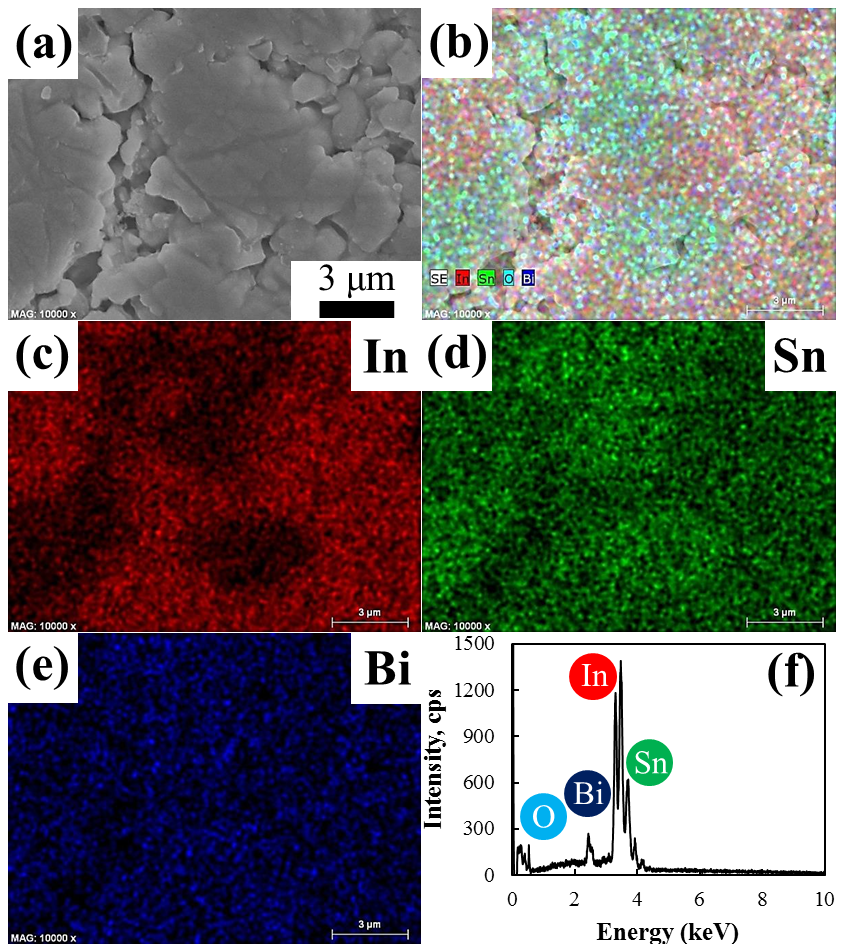
**

**Figure S4.** Cross-sectional SEM image (a), mapping analysis results (b–e), and EDS analysis of the In–Sn–Bi alloy (f) with In L_α_ (3.286 keV), Sn L_α_ (3.443 keV), Bi M (2.419 keV), and O K_α_ (0.525 keV).

**
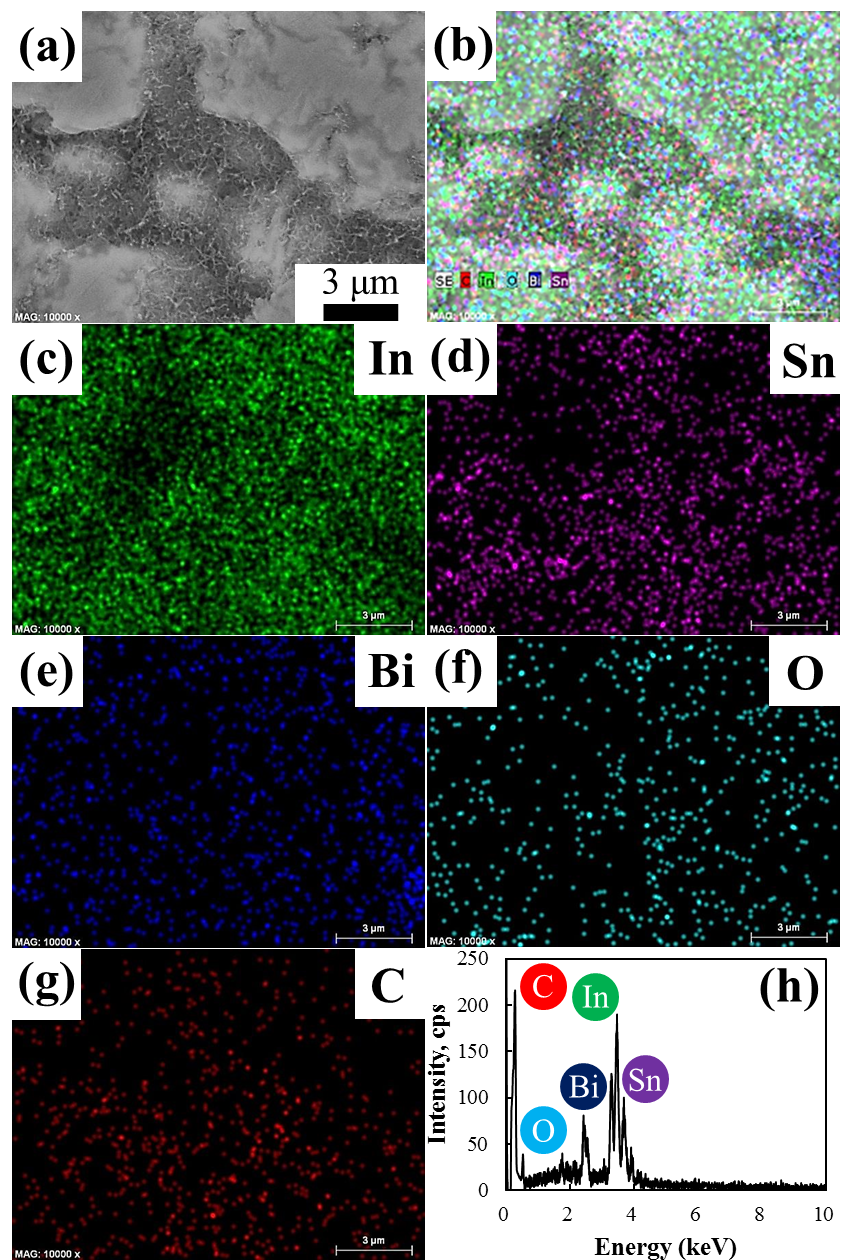
**

**Figure S5.** Cross-sectional SEM image (a), mapping analysis results (b–g), and EDS analysis of 1.2MWCNT/In–Sn–Bi composite (h) with In L_α_ (3.286 keV), Sn L_α_ (3.443 keV), Bi M (2.419 keV), O K_α_ (0.525 keV), and C K_α_ (0.277 keV).

**
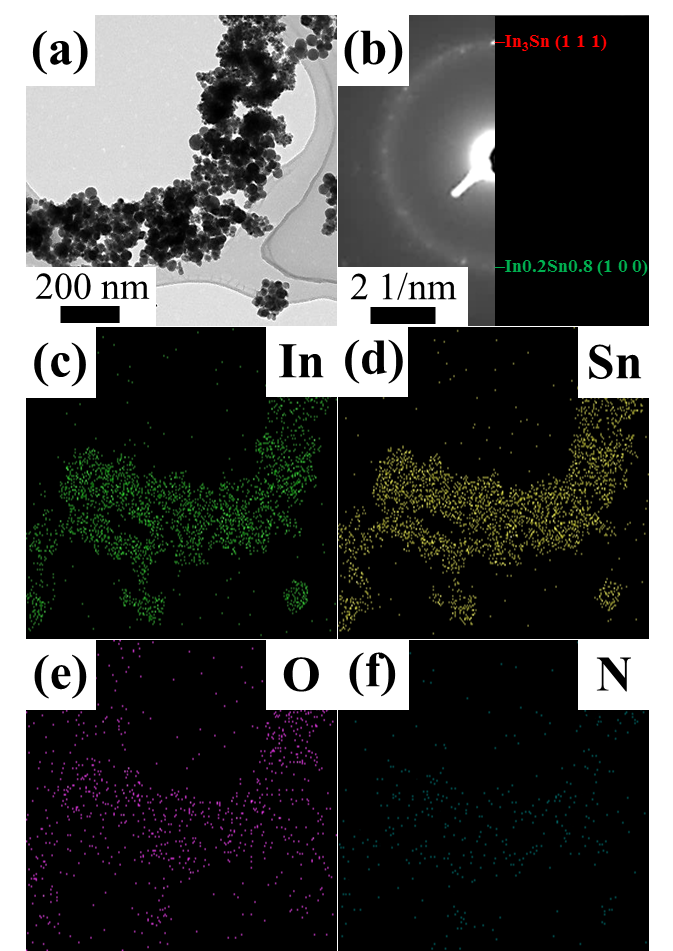
**

**Figure S6.** Low magnification TEM image (a), SAED pattern (b), and mapping analysis (c–f) of In–Sn nanoparticles: In of (c), Sn of (d), O of (e), and N of (f).


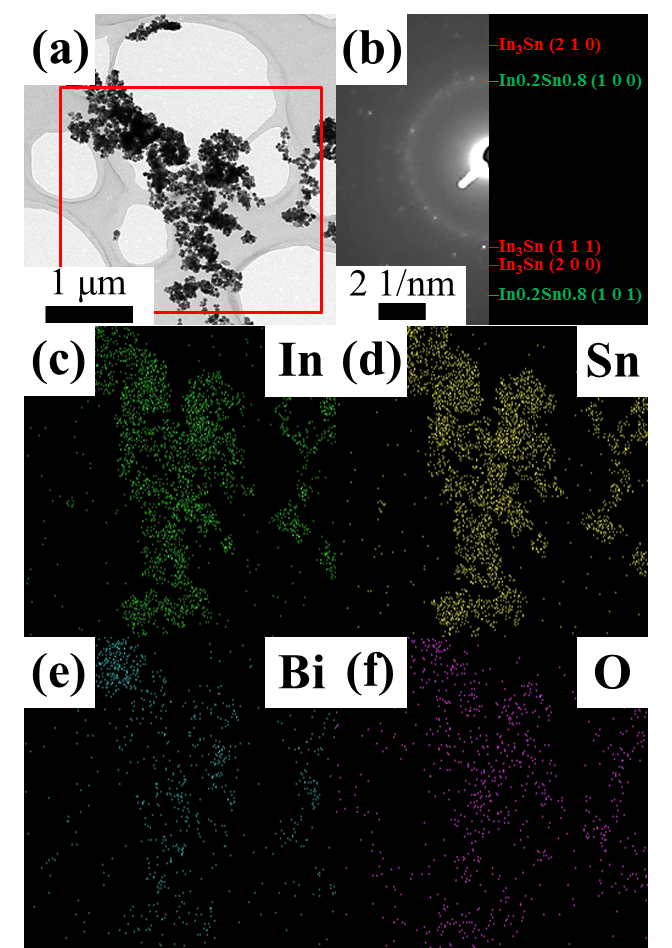


**Figure S7.** Low magnification TEM image (a), SAED pattern (b), and mapping analysis (c–f) of In–Sn–Bi nanoparticles: In of (c), Sn of (d), Bi of (e), and O of (f).

**
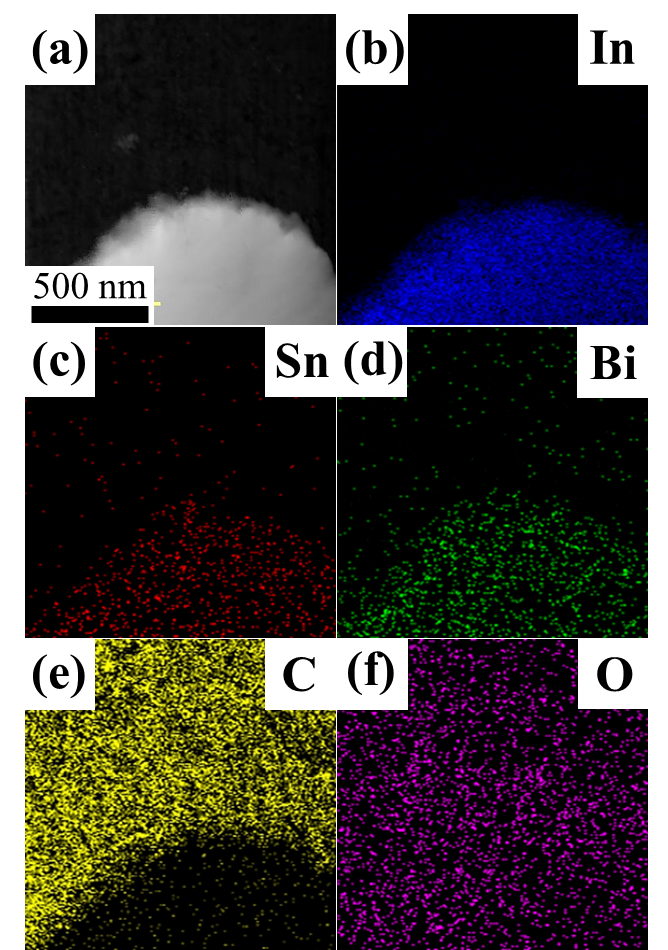
**

**Figure S8.** High-angle annular dark-field (HAADF) image (a) and mapping analysis (b–f) of 1.2MWCNT/In–Sn–Bi composite solder: In of (b), Sn of (c), Bi of (d), C of (e), and O of (f).

**
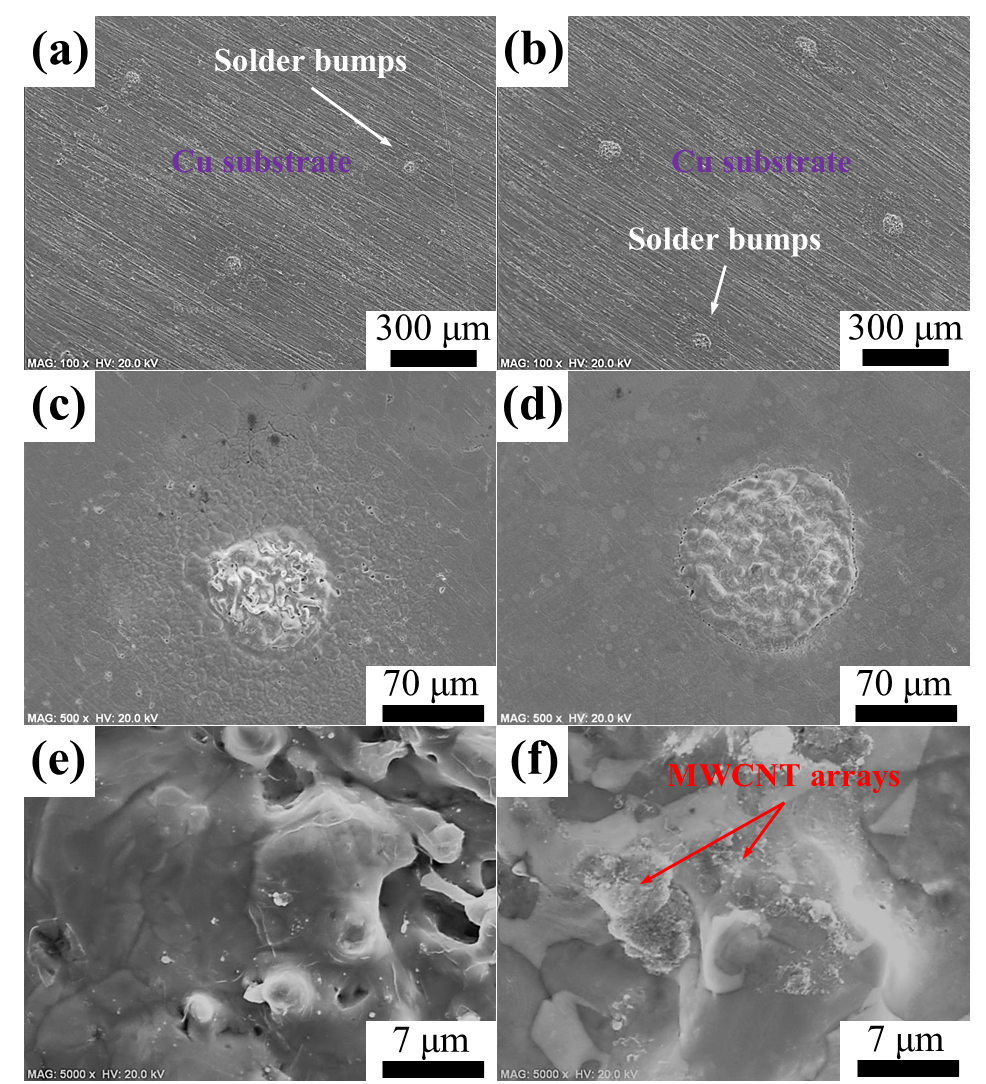
**

**Figure S9.** SEM images of the In–Sn–Bi conventional solder bumps (a, c, and e) and the 0.6MWCNT/In–Sn–Bi composite solder bumps (b, d, and f) reflowed on the Cu substrate at 110 °C. The composite solder bumps had wider and more uniform dispersion on the Cu substrate than the conventional solder bumps due to their improved diffusivity and wettability induced by the MWCNT arrays.


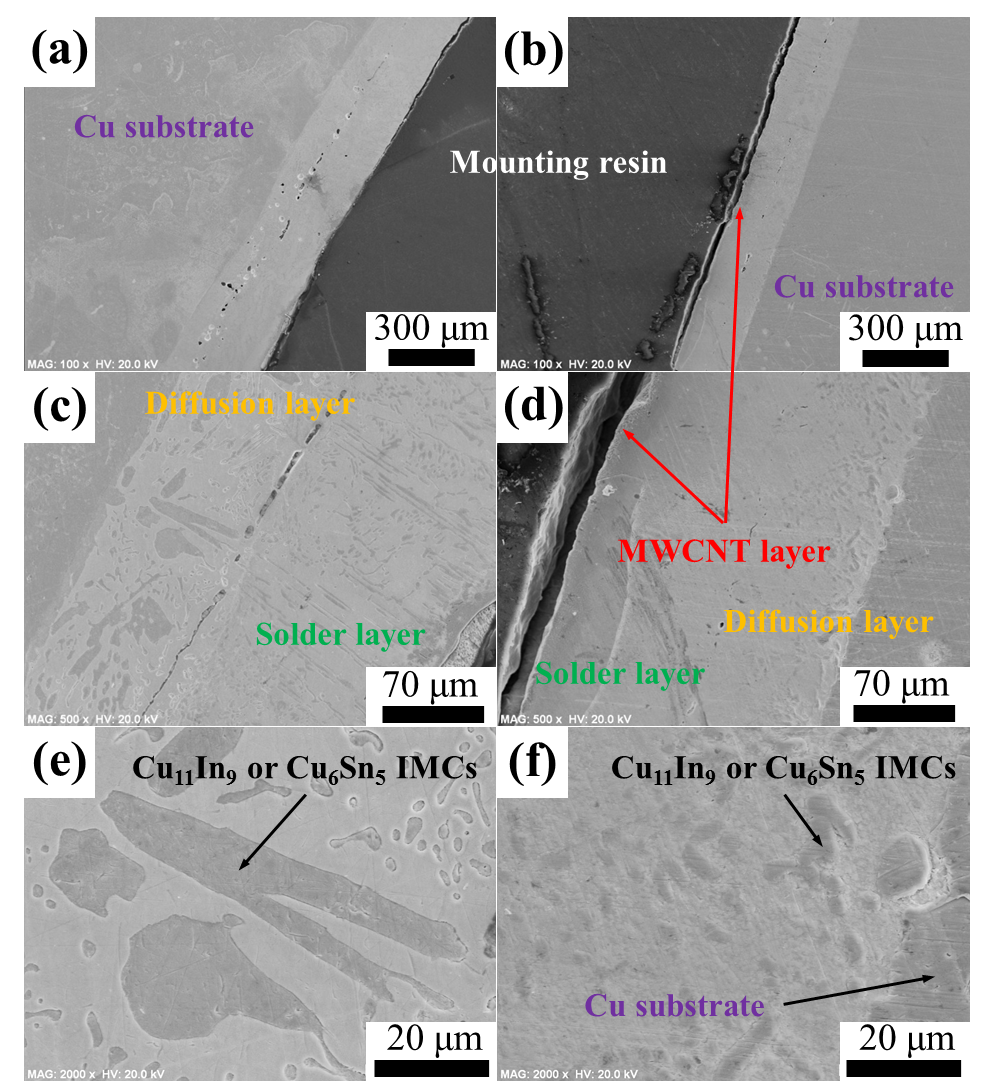


**Figure S10.** Cross-sectional SEM images of the In–Sn–Bi conventional solder (a, c, and e) and the 0.6MWCNT/In–Sn–Bi composite solder (b, d, and f) reflowed on the Cu substrate at 110 °C. There were more micro voids and cracks between the solder and diffusion layers with the conventional solder than with the composite solder due to the abundant formation of brittle IMCs caused by the active diffusion of the solder toward the Cu substrate. For the composite solder, the formation of the IMCs was interrupted by the suppression effect caused by the MWCNT arrays. The MWCNT layer present in the top layer of the composite solder was mostly removed by the polishing and etching process.


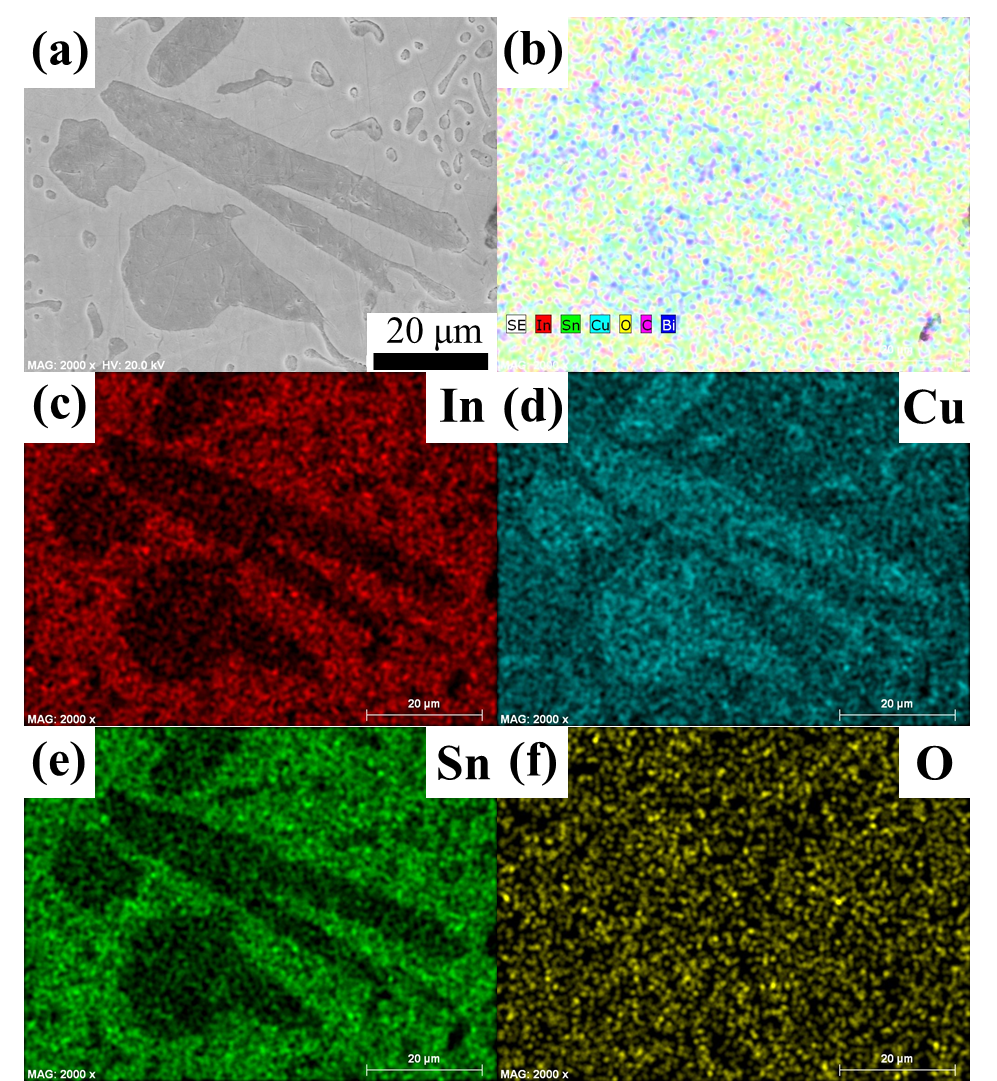


**Figure S11.** Cross-sectional SEM image and EDS mapping images of the diffusion layer of the In–Sn–Bi conventional solder on the Cu substrate. It exhibited abundant Cu–In and Cu–Sn phases compared with those of the 0.6MWCNT/In–Sn–Bi composite solder.


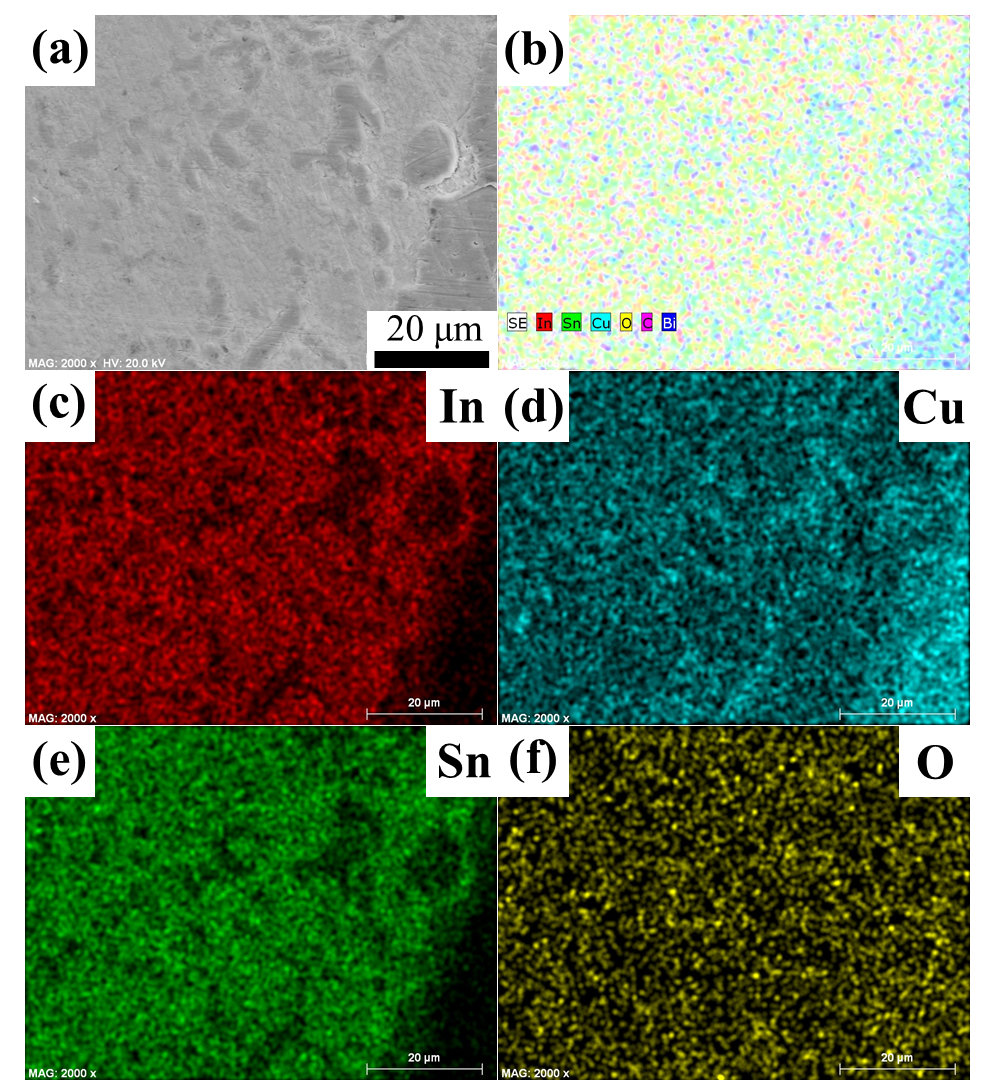


**Figure S12.** Cross-sectional SEM image and EDS mapping images of the diffusion layer of the 0.6MWCNT/In–Sn–Bi composite solder on the Cu substrate. It exhibited less Cu–In and Cu–Sn phases than those of the In–Sn–Bi conventional solder due to the suppression effect by the MWCNT arrays during the IMC formation.


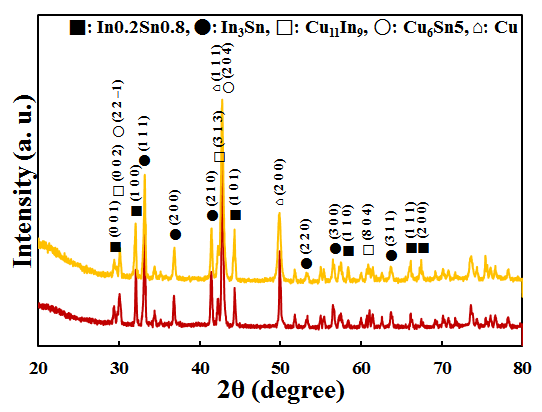


**Figure S13.** XRD patterns of the In–Sn–Bi conventional solder and the 0.6MWCNT/In–Sn–Bi composite solder on the Cu substrate. Their diffusion on the Cu substrate induced new IMC formation, including Cu_11_In_9_ and Cu_6_Sn_5_, along with pre-existing IMCs and the Cu substrate, such as In_3_Sn, In0.2Sn0.8, BiIn, and Cu.
